# Single-Cell RNA-Sequencing Atlas Reveals the Tumor Microenvironment of Metastatic High-Grade Serous Ovarian Carcinoma

**DOI:** 10.3389/fimmu.2022.923194

**Published:** 2022-07-22

**Authors:** Yingqing Deng, Yuan Tan, Dongmei Zhou, Youhuang Bai, Ting Cao, Caizhou Zhong, Weilai Huang, Yuhua Ou, Linlang Guo, Qianqian Liu, Deling Yin, Lipai Chen, Xiping Luo, Deqiang Sun, Xiujie Sheng

**Affiliations:** ^1^ Key Laboratory of Biological Targeting Diagnosis, Therapy and Rehabilitation of Guangdong Higher Education Institutes, The Fifth Affiliated Hospital of Guangzhou Medical University, Guangzhou, China; ^2^ Department of Cardiology of The Second Affiliated Hospital, Cardiovascular Key Laboratory of Zhejiang Province, School of Medicine, Zhejiang University, Hangzhou, China; ^3^ Department of Obstetrics and Gynecology, Center for Reproductive Medicine/Department of Fetal Medicine and Prenatal Diagnosis/BioResource Research Center, Key Laboratory for Major Obstetric Disease of Guangdong Province, The Third Affiliated Hospital of Guangzhou Medical University, Guangzhou, China; ^4^ School of Life Science and Technology, Tongji University, Shanghai, China; ^5^ Department of Research and Development, Zhejiang Gaomei Genomics, Hangzhou, China; ^6^ Department of Obstetrics and Gynecology, Guangdong Women and Children Hospital, Guangzhou, China; ^7^ Department of Pathology, Zhujiang Hospital, Southern Medical University, Guangzhou, China; ^8^ Affiliated Cancer Hospital and Institute of Guangzhou Medical University, Guangzhou, China

**Keywords:** scRNA-seq, tumor microenvironment, T cells, myeloid cells, high-grade serous ovarian carcinoma

## Abstract

Ovarian cancer is the most common and lethal gynecological tumor in women worldwide. High-grade serous ovarian carcinoma (HGSOC) is one of the histological subtypes of epithelial ovarian cancer, accounting for 70%. It often occurs at later stages associated with a more fatal prognosis than endometrioid carcinomas (EC), another subtype of epithelial ovarian cancer. However, the molecular mechanism and biology underlying the metastatic HGSOC (HG_M) immunophenotype remain poorly elusive. Here, we performed single-cell RNA sequencing analyses of primary HGSOC (HG_P) samples, metastatic HGSOC (HG_M) samples, and endometrioid carcinomas (EC) samples. We found that ERBB2 and HOXB-AS3 genes were more amplified in metastasis tumors than in primary tumors. Notably, high-grade serous ovarian cancer metastases are accompanied by dysregulation of multiple pathways. Malignant cells with features of epithelial-mesenchymal transition (EMT) affiliated with poor overall survival were identified. In addition, cancer-associated fibroblasts with EMT-program were enriched in HG_M, participating in angiogenesis and immune regulation, such as IL6/STAT3 pathway activity. Compared with ECs, HGSOCs exhibited higher T cell infiltration. PRDM1 regulators may be involved in T cell exhaustion in ovarian cancer. The CX3CR1_macro subpopulation may play a role in promoting tumor progression in ovarian cancer with high expression of BAG3, IL1B, and VEGFA. The new targets we discovered in this study will be useful in the future, providing guidance on the treatment of ovarian cancer.

## Introduction

Epithelial ovarian cancer usually occurs at an advanced stage and is the most common cause of death from gynecological cancer (1). High-grade serous ovarian carcinoma (HGSOC) is a common histological subtype of epithelial ovarian carcinoma, for 70 to 80%, while the endometrioid carcinoma (EC) subtype accounts for 10% (2). HGSOC is associated with a more fatal prognosis and frequent recurrences, with more than 85% of women with this type having a 10-year mortality rate of 70%, whereas endometrioid carcinoma is thought to originate in endometriosis, a histologic type that tends to have a better prognosis. Our knowledge of the molecular etiology and clinical pathology of HGSOC has greatly improved, and recent therapy has advanced (3–6). However, most patients are diagnosed at a late stage, when cancer has already metastasized, and the diagnosis results in 5-year survival of 30%. Accordingly, the development of effective therapies for metastatic ovarian cancer is urgently needed. To do so, we need to comprehensively characterize the cellular heterogeneity and define transcriptional features within the tumor microenvironment.

Genomic analysis of HGSOC revealed a mutation in the tumor suppressor gene TP53, which is also seen in endometrioid carcinoma (3), promoting ovarian cancer metastasis and chemoresistance, and defecting in homologous recombination (HR) DNA repair, which contributes to the somatic BRCA mutation (7). The Cancer Genome Atlas (TCGA) project has classified HGSOC into four transcriptional subtypes: ‘differentiated’, ‘immunoreactive’, ‘proliferative’, and ‘mesenchymal’ (8, 9).

Recently, a scRNA-seq study of six metastatic omental tumors that derived from primary HGSOCs unraveled the genetic signatures of immune cell subsets within the tumor microenvironment and identified NR1H2^+^ IRF8^+^ and CD274^+^ macrophage clusters, which were suggested with an anti-tumor response (10). Another scRNA-seq study revealed that the inhibition of the JAK/STAT pathway has potential anti-tumor activity (11). The heterogeneity of tumor cells and different immune cell types within the TME play a paramount role in shaping tumor behavior (12–14). Therefore, characterizing the complex interplay between tumor cells and immune cell phenotype within HGSOC will be beneficial to find the critical factors of ovarian carcinogenesis, metastasis, and targeted treatment.

In this study, we conducted single-cell RNA sequencing of five primary high-grade serous carcinomas samples (HG_P), three metastases from HGSOC to the peritoneum (HG_M), one normal ovarian sample, and two primary Endometrioid (EC_P) samples. By comparing HG_M with HG_P and EC_P, we comprehensively characterized the heterogeneity of tumor cells and immune cells in ovarian cancer lesions, as well as the dynamic changes in cell-type composition and intercellular interactions, providing new insights into the biological basis of the development of HGSOCs and ECs.

## Results

### Single-Cell Transcriptomic Profiling of the Cellular Heterogeneity of the HGSOCs

**Figure 1:**
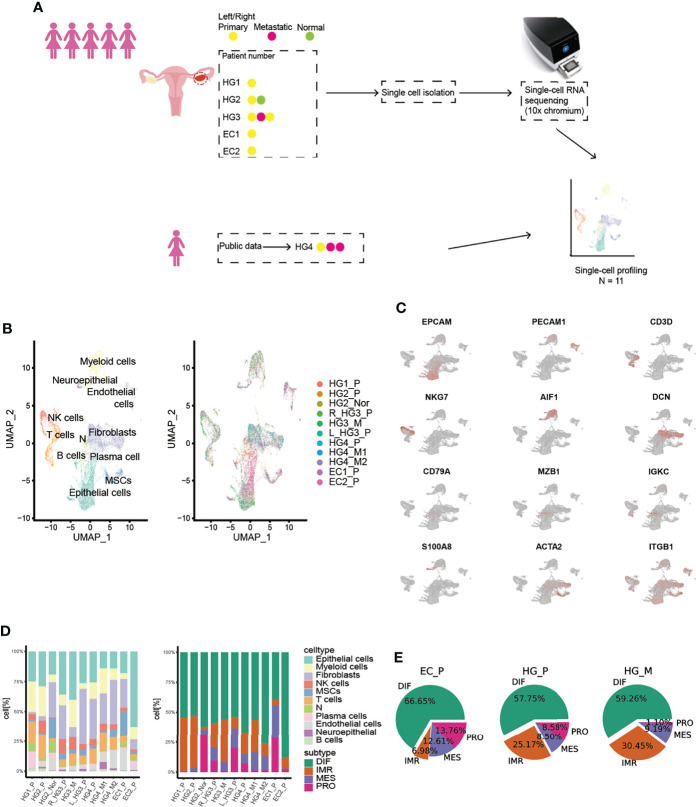
Overview of TME in primary HGSOCs, metastatic HGSOCs, and ECs. **(A)** Workflow of the samples collected and the data analysis strategy. **(B)** Cell populations identified. The UMAP projection of 55802 single cells from HG_P (n=5), HG_M (N=3), EC_P (n=2), HG_Nor (n=1) samples shows the 10 main clusters with annotation. Each dot corresponds to a single cell, colored according to cell type. **(C)** Canonical cell markers are used to identify the clusters. **(D)** Barplots of the cell type and cancer subtypes for all 11 tumors. **(E)** The cancer subtypes proportion for each pathological group.

Droplet-based single RNA-seq (10X Genomic) was performed on a total of eight samples from five treatment-naive patients. For parallel analyses, the public scRNA-data of 1 HG_P and 2 HG_M samples (15) from the same patient were downloaded (**Figure 1A**, **Tables S1**, **S2**). After quality filtering, approximately 0.68 billion unique molecular identifiers (UMIs) were collected from 55802 cells with >250 genes detected. Of these cells, 28,571(51.2%) cells were from HG_P, 8925 (16%) cells were from HG_M, and 12751(22.9%) cells were from EC_P. All high-quality cells were used to perform canonical correlation analysis (CCA) and identify anchors or mutual nearest neighbors (MNNs). Then, we integrated all cells, conducting unsupervised graph-based clustering (16).

By Uniform Manifold Approximation and Projection (UMAP) with the resolution of 1.1, we identified 10 major lineages (epithelial cells, B cells, NK cells, T cells, plasma cells, fibroblasts, mesenchymal stem cells (MSCs), endothelial cells, neutrophils, and myeloid cells) (**Figures 1B, C**, **S1A**). The cell types were mainly assigned based on canonical cell markers and functional categories according to significantly differential genes expressed from different clusters (17, 18). One remaining cluster was labeled as “N” because we could not confidently recognize this cell type (10). The respective proportion of each cell type was varied and significantly differed between tumors (**Figure 1D**, proportion test, df=10, p-value < 2.2e-16). Interestingly, the boxplot exhibited that the medians in B cells, NK/T cells, and myeloid cells were higher in HG_P than HG_M and EC_P, whereas plasma cells were more enriched in EC_P (**Figure S1B**).

Previously, The Cancer Genome Atlas (TCGA) had stratified HGSOC into four molecular subtypes. We wondered if the inter-patient variability among tumors were consistent with these subtypes. To this end, we assigned molecular subtypes to our samples with the consensusOV (9) classifier (Version 1.14.0) (**Figure 1D**, **S2A**). We found that all four subtypes were well presented in each ovarian lesion. EC_P expressed the DIF signature slightly over HG_P and HG_M, while HG_P was comparable with HG_M, and EC_P presented the lowest IMR signature, supporting low immune-cell infiltration (**Figure 1E**). What’s more, certain subtypes tended to be consistent with specific cell types (**Figure S2B**). Epithelial cells highly expressed the differential (DIF) signature and lesser expressed proliferative (PRO) signature. The mesenchymal (MES) signature was strongly expressed by the fibroblasts and MSCs cells, while the immunoreactive (IMR) signature mainly consisted of myeloid, T cells, and NK cells. Notably, more fibroblasts were classified as proliferative subtypes in our data, suggesting that there are more fibroblasts with relatively high tumor purity. Based on this result, we wanted to know which genes made the most contribution to this classification. Thus, we extracted markers that were used for classification and calculated their average expression in each cell type (**Figure S3A**). As expected, proliferative markers such as MARCKSL1, STMN1, UCHL1, MFAP2, TRO, etc. are indeed expressed at higher levels in fibroblasts than others. Likewise, we plotted these markers in the heatmap in each cell of fibroblasts (**Figure S3B**) and we found PRO-markers like MARCKSL1 and STMN1, especially for MARCKSL1, which are highly expressed in most cells. And these markers contribute the most to the classifications of proliferative-subtype, while other PRO-related markers, UCHL1 and MFAP2, are not expressed significantly per cell in fibroblasts though their average expression is higher than other cell types. These results illustrated the importance of fibroblasts in cancer progression and indicated that a subset of fibroblasts in our data is cancer-associated fibroblasts (CAFs).

In addition, we can assess TCGA-subtypes at the patient level from our single-cell data by calculating the average of each gene per sample. Validated with the dataset from a previous study (19) (**Figure S4A-4C**), we had a high degree of confidence to infer the subtypes of HG1_P, HG2_P, HG4_M2, and EC2_P (**Figure S4D, 4E**). Their subtypes are likely to be IMR, IMR, MES, and DIF. Overall, our findings fully illustrated the difference in subtype classification between bulk samples and single-cell data, where single cells can more accurately describe the TCGA-subtype and characterize the heterogeneity of ovarian cancer.

### Distinguish Worse Survival Cells from Cancer Epithelial Cells of HGSOCs

**Figure 2:**
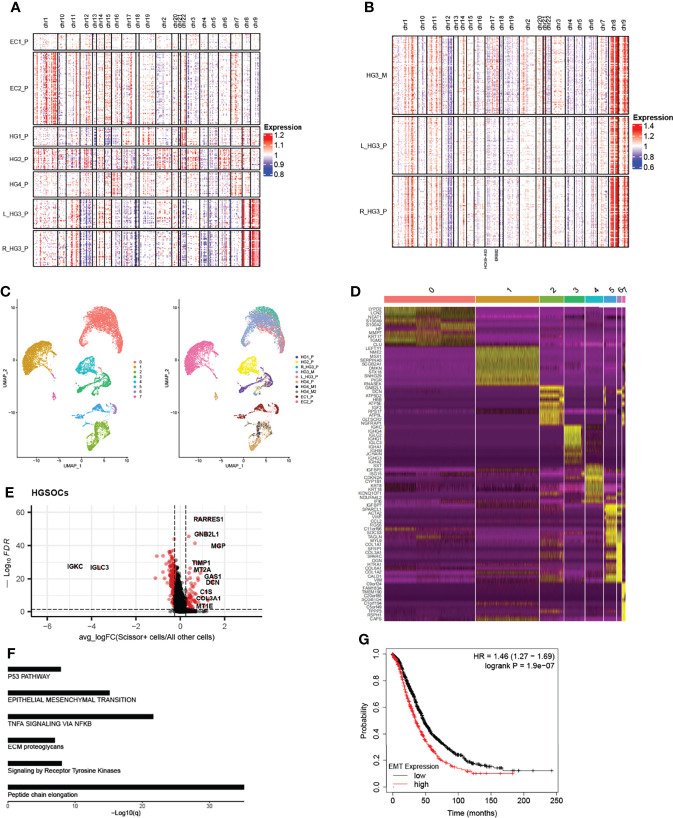
Copy number profiles, intertumoral heterogeneity, and EMT signature subpopulations are identified. **(A)** The chromosomal landscape of copy number for 13,634 epithelial cells of seven primary tumors; amplification (red) and deletions (blue). **(B)** The chromosomal landscape of copy number for 2849 epithelial cells of metastatic tumors and primary tumors of the HG3 patient (L_HG3_P means the primary tumor from the left ovary in the HG3 patient; R_HG3_P means the primary tumor from the right ovary in HG3 patient). **(C)** The UMAP projection of 17,551 epithelial cells from 10 tumors of six patients (indicated by labels and colors) reveals tumor-specific clusters. **(D)** Differentially expressed genes of the top 10 genes (rows) that are differentially expressed in each cluster (columns). **(E)** Differentially expressed genes between Scissor^+^ cells and all other cells in HGSOCs, each point represents a gene. Red: significant genes; Black: NS genes. avg_logFC: log 2 fold-change of the average expression between the two groups. ((log-FC > 0.25, FDR <0.05) **(F)** Enrichment of significant genes related to Reactome and Hallmark pathways. **(G)** Kaplan-Meier plot shows that high expression of EMT signature has shorter overall survival in ovarian cancer. The high and low patients are split by the mean expression of the EMT-related gene set.

Based on the expression of PAX8 and CD24 (20, 21), we found that they were mainly expressed in epithelial cells as well as the subtype DIF (**Figures S2A, S5A**), which we termed “cancer epithelial cells”. In addition, we used T cells and myeloid cells from the normal sample (HG2_nor) as controls by inferring chromosomal copy number alterations (InferCNV, Version 1.2.1) (11) to confirm this (**Figure S6**). As shown below, we found that CNV trends in the same chromosomal region of different primary patients were distinct, whereas they were approximately consistent in the same patient (**Figures 2A, B**, **S5B**). Obviously, the genes generally mutated in ovarian cancer were more amplified in chr17 in HG3_M than in the primary tumors on both sides, such as the ERBB2 and HOXB-AS3 genes, which are generally mutated in ovarian cancer (8). These results demonstrated both intertumoral heterogeneity between patients and consistency within the same patient lesion.

Re-running UMAP analysis on these cells, a total of eight sub-clusters were identified, of which revealed interpatient tumor-specific clusters (22, 23) (**Figure 2C**). Conversely, re-clustering the subsets like T cells and fibroblast cells without integration, we found that ovarian lesions from the same batch clustered together (**Figure S5C**). Differentially expressed genes in each cluster, interpatient GSVA, and cell cycle analysis were also shown (**Figures 2D** and **S5D, 5E**). We then used a Scissor algorithm (Version 2.0.0) to classify cells associated with worse survival (Scissor^+^ cells) from cancer epithelial cells, based on GDC TCGA bulk RNA-seq expression and clinical phenotype (24). As described previously, Scissor can quantify the similarity between single-cell data and bulk data through measurements, for example, Pearson correlations, and then it optimizes a regression model on the correlation matrix with the sample clinical phenotype. After that, it will provide feedback on three cell types, such as cells associated with worse prognosis (Scissor^+^ cells), cells related to better prognosis (Scissor^-^ cells), and cells that have no relationship with prognosis (Background cells).

In HGSOCs, we found that Scissor^+^ epithelial cells mainly accumulated in patients with metastasis (**Figure S7A**). To distinguish Scissor^+^ cells from Scissor^-^ cells and Background cells (All other cells), we compared their gene expression (**Figure 2E**, **S7C**, cut off: avg_log-FC > 0.25, FDR <0.05). Interestingly, high expression of EMT-related genes like GAS1, DCN, COL1A1, MGP, etc. in HGSOCs Scissor^+^ cells derived predominantly from patients with tumors metastasized (**Figures 2E**, **S7B**). Genes such as STMN1, CCND1, TUBA1B, and TUBB significantly expressed in Scissor^+^ cells of ECs were associated with the cell cycle (**Figure S7E**), which is a barometer of epithelial tumor cells proliferation (25). Furthermore, functional enrichment analysis of significantly expressed genes in Scissor^+^ cells also confirmed these (**Table 1**, **Figures 2F**, **S7D**), and survival analyses revealed that high levels of EMT and cell cycle signature were significantly related to poor overall survival in the Ovarian Cohort (**Figures 2G**, **S7F**). The observation of HGSOCs was consistent with the previous report that EMT is involved in increasing the invasion and metastasis of epithelial tumors (26–29). Therefore, targeting epigenetic regulation of EMT is a potentially powerful approach to inhibit the migration and invasiveness of HGSOCs.

**Table 1:** Functional enrichment analysis based on the upregulated genes in HGSOCs Scissor^+^ cells versus All other cells (Scissor^-^ and Background cells).

Pathological subtype	Category	Description	Log10(q)	Genes
HGSOC	Reactome Gene Sets	Peptide chain elongation	-35.94	COL3A1, MT1E, MT2A, ZFP36, FOS, HBB, RPS17, NNMT, RPL13A, COL6A2, COL6A1, RPS9, JUN, RPL31, SLC25A6, RPL34, SAT1, RPS4X, ZFP36L1, RPS6, EEF2, RPS5, RPL11, RPL13, RPL23, CEBPB, RPL10A, RPS12, RPS20, RPL19, RPL10, RPS16, RPLP2, ID1, ACTB, RPL18, RPS14, RPS3, RPL7
HGSOC	Reactome Gene Sets	Signaling by Receptor Tyrosine Kinases	-8.00	COL3A1, COL1A1, FOS, JUNB, ID3, COL6A2, EGR1, FOSB, LAMA4, NR4A1, COL6A1, MYC, FN1, ID1, ACTB
HGSOC	Reactome Gene Sets	ECM proteoglycans	-6.99	DCN, COL3A1, COL1A1, C3, TIMP1, HBB, COL6A2, HP, HTRA1, LAMA4, COL6A1, FN1, FTL
HGSOC	Hallmark Gene Sets	HALLMARK_TNFA_SIGNALING_VIA NFKB	-21.61	DCN, MT1E, MT2A, ZFP36, FOS, JUNB, EGR1, DUSP1, FOSB, CYR61, NR4A1, PNRC1, MYC, IER2, JUN, KLF4, SAT1, NR4A2, IER3, CEBPB, KLF6, EIF1, UGP2
HGSOC	Hallmark Gene Sets	HALLMARK_EPITHELIAL_MESENCHYMAL TRANSITION	-15.12	MGP, GAS1, DCN, COL3A1, COL1A1, TIMP1, VIM, NNMT, COL6A2, CYR61, HTRA1, JUN, FN1, SAT1, IGFBP4
HGSOC	Hallmark Gene Sets	HALLMARK_P53_PATHWAY	-7.88	GNB2L1, FOS, JUN, KLF4, SAT1, ZFP36L1, IER3, RPS12, RPL18, ISCU

### Dynamic Trajectory Analysis During the Progression of HGSOCs

**Figure 3:**
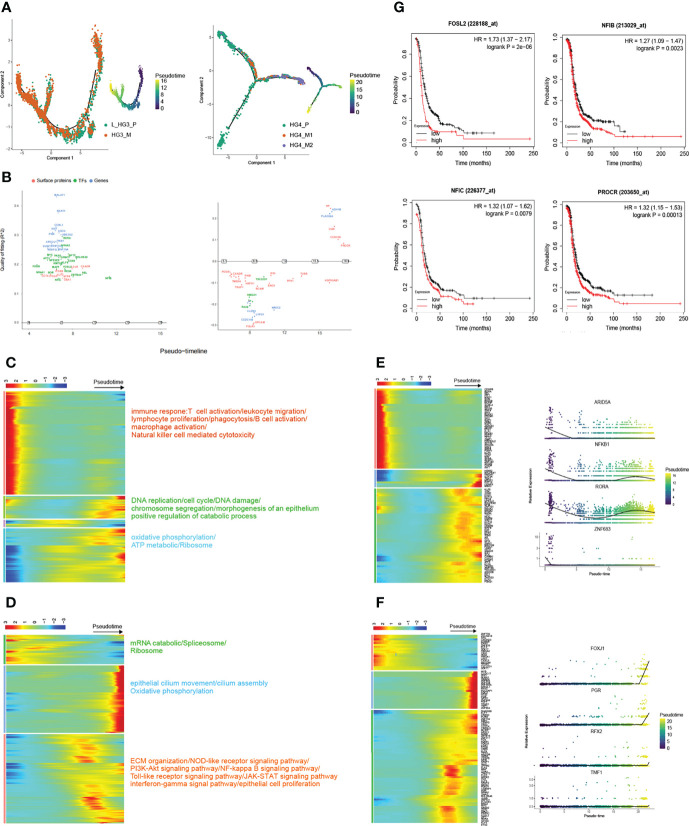
Trajectory reconstruction during metastatic HGSOCs. **(A)** Monocle2 infers the development of epithelial cells along with pseudo-time (from patients HG3 and HG4 respectively, L_HG3_P means the primary tumor from the left ovary). Pseudo-time legend from dark to bright indicates cancer progression from the early to late stage. **(B)** Genswitches deduces the genes switch between cell states (left: L_HG3; right: HG4). **(C**, **D)** The heatmap displays the dynamic gene expression profiles during metastasis of ovarian cancer (from patients HG3 and HG4 respectively). The color key from blue to red indicates relative expression levels from low to light. The top annotated GO and KEGG terms in each cluster are shown. **(E**, **F)** Top 100 differentially expressed transcription factor genes (TFs; left) and the expression of specific TFs are on view along with the pseudo-time curve in (right). **(G)** Overexpression of proliferation and metastasis-related genes predicts poor prognosis in HGSOCs.

In the recent past, omentum metastasis has been reported (10). But in general, there are still few studies on the genetic dynamics of high-grade serous ovarian cancer metastasis, especially on the transcription factors involved in tumor progression. Based on the Monocle2 method (Version 2.21.1), pseudo-time reconstruction of epithelial cells was performed to infer the progression path of HGSOC (**Figure 3A**, and **S8A**). Macroscopically, the number of metastatic epithelial cells increased along the trajectory at the later stage. Besides, we also estimated the RNA velocities of every single cell by distinguishing un-spliced and spliced mRNAs, a function provided by velocyto (30) package. According to the direction of movement of each cell, the process of metastasis of HGSOCs can be clearly detected (**Figure S8D**).

In particular, the dynamic gene expression profiles during the development of tumors were extracted. (**Figures 3C, D**). Interestingly, we found that the molecular mechanisms involved in metastatic HGSOCs were consistent whether the primary HGSOC was on the left or right side (**Figures 3C**, **S8E**). Genes related to the immune response were significantly decreased, whereas the genes related to DNA replication, cell cycle, epithelial cell proliferation, oxidative phosphorylation, and TCA cycle were significantly increased (**Figures 3C, D**). These results also suggested that inhibitors based on poly (ADP-ribose) polymerase (PARP), which aids in stopping the regeneration of cancer cells, such as Olaparib and Rucaparib, may be helpful for the treatment of metastatic HGSOCs (31–33). Meanwhile, the transcriptional factors (TFs) related to immune regulation, such as ARID5A, NFKB1, RORA, and ZNF683, were gradually downregulated along with the trajectory differentiation process (**Figure 3E**). And these immune-related TFs were scattered in the clusters of epithelial cells as well as other cell types (**Figure S8B**). Conversely, some well-known factors related to tumor growth promotion, such as HMGA1, GTF3A, PHF19, CENPX, and MBD2, were upregulated. Zingg emphasized that loss of cilia accelerates melanoma metastasis in benign cells by enhancing Wnt/β-Catenin Signaling (34). In our data, the expression of epithelial cilium movement markers, including FOXJ1, PRG, RFX2, and TMF1, although increased during the process, were mainly overexpressed in primary tumor cells (**Figures 3A, F**). This may indicate that disruption of cilia assembly leads to primary ovarian cancer that metastasizes to the peritoneum.

We also utilized GeneSwitches (35) (Version 0.1.0) to predict the genes that act as on/off switches between cell states in order during the tumor’s metastasis process (**Figures 3B**, **S8C**). Accordingly, overexpression of some acting on genes like FOSL2, NFIB, NFIC, and PROCR, which are associated with proliferation and metastasis, predicts poor prognosis in high-grade serous ovarian cancer (**Figure 3G**). Taken together, these results reveal dynamic gene expression profiles, highlighting several quintessential TFs and surface proteins that are dysregulated during ovarian cancer progression.

### Cancer-Associated Fibroblasts with the EMT Program Enriched in Metastatic HGSOCs

**Figure 4:**
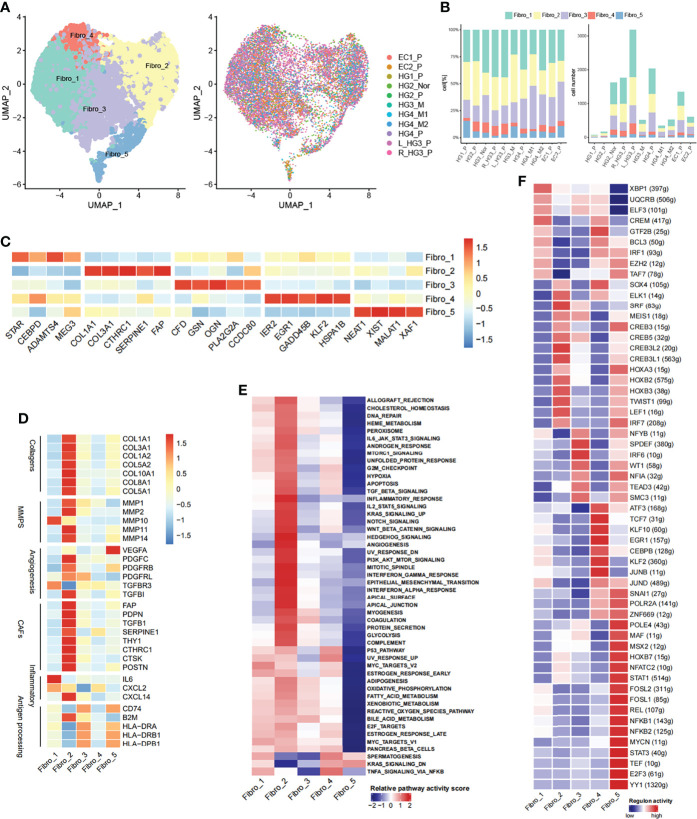
Diversity of fibroblasts in HG_M. **(A)** The UMAP projection of 12,236 fibroblast cells of 11 samples from six patients (indicated by labels and colors). **(B)** Proportion and cell number of each fibroblast subtype in 11 samples. **(C)** Heatmap of marker genes expression. **(D)** Heatmap of functional gene sets. **(E)** GSVA analysis of differential pathways is scored per cell among five fibroblast subsets. **(F)** Active regulons in each fibroblast subsets.

Fibroblast is another vital biological cell type that synthesizes the extracellular matrix and collagen to maintain the structural integrity of connective tissue. In this study, 12,236 fibroblast cells were categorized into five distinct sub-clusters (**Figures 4A**, **S9B, 9C**). Fibro_1 cells were marked by STAR, an exclusive marker for ovarian stromal cells as previously reported (15). Fibro_2 cells were characterized by collagen (COL1A1, COL3A1) and cancer-associated fibroblast genes (CTHRC1, FAP). Fibro_3 cells expressed immunomodulatory (CFD, OGN) and tumor suppressor genes (CCDC80, PLA2G2A). Fibro_4 cells expressed growth factors (EGFR, IER2M KLF2). Fibro_5 cells were characterized by conserved and nuclear-enriched lncRNA (MALAT1, NEAT1), and MALAT1 modulates the expression of cell cycle-related genes in lung fibroblast and EMT-related genes in breast cancer (36, 37) (**Figure 4C**). The distribution of fibroblast sub-clusters in each tumor was varied (**Figure 4B**). In addition, comparisons based on hallmark gene sets of fibroblasts had been conducted. Fibroblasts from HG_M were more abundant in supporting tumor progress than HG_P, including angiogenesis, coagulation system, and EMT (**Figure S9D**). Fibroblasts from EC_P were more enriched in the structure formation of tissue than HG_P, such as myogenesis and adipogenesis, whereas inflammatory pathways including TNFA signaling *via* NFκB and inflammatory response more enriched in HG_P than EC_P (**Figure S9E**). Combined with the analysis of normal fallopian tubes (nFT) from previous studies (38, 39), we found that fibroblasts from HG_P were more abundant in supporting interferon-alpha/gamma response than HG_nor/nFT while estrogen responses early activity was more active in HG_nor/nFT than HG_P (**Figure S9F**).

Notably, Fibro_2 cells, the most enriched subtype in HG_M, accounting for 32%, expressed genes of cancer-associated fibroblasts (CAFs), angiogenesis, and collagen at a high level (**Figures 4D**, **S9A**). CAFs have been verified to promote tumor metastasis through upregulating genes like HSF1, which was involved in the pro-tumorigenic pathway (40). Furthermore, Hallmark pathway analysis also confirmed that Fibro_2 cells had more relevance with the pathways that sustain tumor growth, including angiogenesis, epithelial-mesenchymal transition (EMT), hypoxia, and PI3K/AKT/mTOR signaling (**Figure 4E**). Compared with HG_M and EC_P, Fibro_3 cells (19%) and Fibro_5 cells (3%) accounted for less proportion in HG_P. Intriguingly, Fibro_3 cells and Fibro_5 cells were both consistent with the characteristic of “antigen-presenting CAFs” as previously discerned (41), owning to express genes like CD74 and human leukocyte antigen (**Figure 4D**).

With the help of the SCENIC tool, we identified regulons unique to each fibroblast sub-clusters (**Figure 4F**). For instance, the transcription factors of SOX4 and SRF underpinned Fibro_2, while Fibro_5 cells were characterized by STAT1/STAT3 and NFKB1/NFKB2. Strikingly, SOX4 has been proven as an important co-factor of SMAD3, controlling pro-metastatic gene transcription and shaping the cell response to TGF-β in different scenarios, thereby promoting tumorigenesis (42). While STAT3 and NF-κB are pro-inflammatory regulators and they form transcriptional complexes that positively regulate gene expression in oncogenic pathways (43).

### Heterogeneity of Tumor-Infiltrating Lymphocytes in HGSOCs

**Figure 5:**
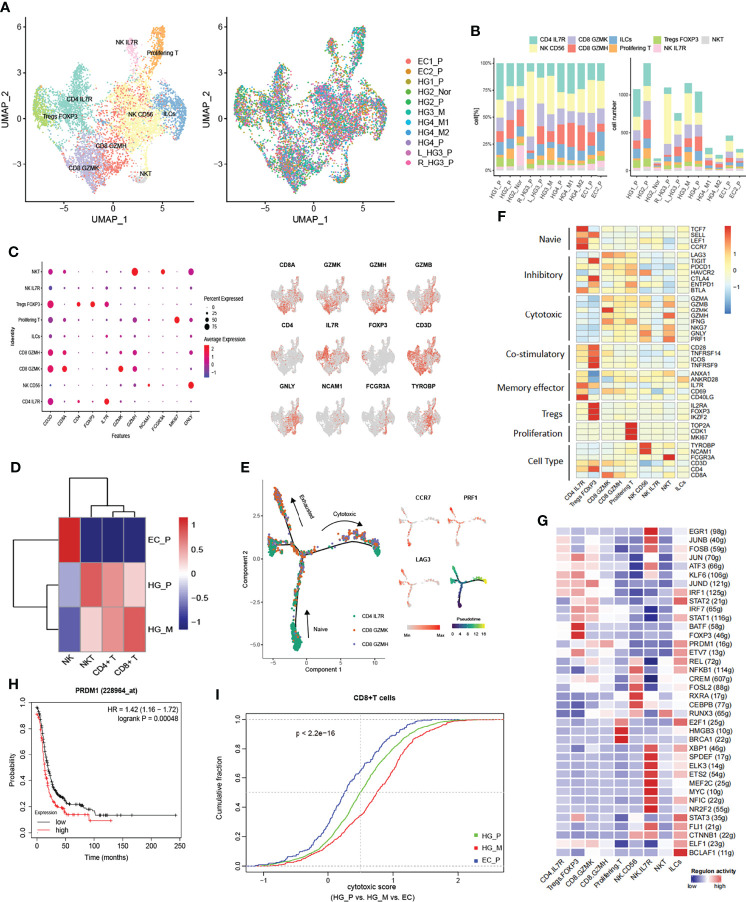
Subpopulations of tumor-infiltrating lymphocytes (TILs) in HG_M. **(A)** The UMAP projection of 7967 TILs of 11 samples from six patients (indicated by labels and colors). **(B)** Proportion and cell number of each subtype in 11 samples. **(C)** Dot plot (left) and UMAP-plot (right) display canonical cell markers. **(D)** Hierarchical clustering heatmap groups the tumors between HG_P, HG_M, and EC_P. **(E)** Reconstruction trajectory of CD8^+^ T cells inferred by Monocle2 (color by subtypes, expression of signature genes, and pseudotime). **(F)** Heatmap of the functional gene sets in TILs. **(G)** Active Regulons in each TILs. **(H)** Overexpression of the PRDM1 gene predicts a worse prognosis in ovarian cancer. **(I)** Cumulative distribution of cytotoxic CD8^+^ T cells between HG_P, HG_M, and EC_P. The cytotoxic score is calculated based on the average expression of cytotoxic markers. P-value was calculated by a two-sided unpaired Kruskal-Wallis rank-sum test.

Infiltration of T cells into tumors modifies the natural course of the disease and plays a critical role in cancer immunotherapy (44, 45). From the ovarian cancer lesion, we classified a total of 7967 T and NK cells into eight subtypes: CD4^+^ T cells (CD4 IL7R; CD3D^+^ CD4^+^), regulatory CD4^+^ T cells (Tregs FOXP3; CD4, FOXP3), CD8^+^ T cells (CD8 GZMK, CD8 GZMH; CD3D^+^ CD8^+^), NK cells (NK CD56, NK IL7R; NCAM1, GNLY, TYROBP, NKG7), NKT cells (CD3D, CD8A, FCGR3A, GNLY), and Innate Lymphoid Cells (ILCs; CD3D) (**Figures 5A, C** and **S10A**). The number of cells and the proportion of each subtype in each tumor were shown (**Figure 5B**). Notably, 1665 T/NK cells were obtained from HG_M, while 5394 T/NK cells were from HG_P. Using a dendrogram to group the tumors based on the average expression of T cell markers, we found that HG_M showed a similar pattern to HG_P, whereas EC_P emerged with low expression in CD4^+^ T and CD8^+^ T cells (**Figure 5D**). This observation was consistent with the boxplot shown, the lower proportion of CD4^+^ T cells and CD8^+^ T cells was detected in EC_P than in HG_P and HG_M (**Figure S10B**). The low tumor-suppressive status in EC_P suggested that T cell-based immunotherapy may be inefficient in EC_P.

Among CD4^+^ T cells, we identified naive (CD4 IL7R; TCF7, CCR7, SELL, LEF1) and regulatory (Tregs FOXP3; IL2RA, FOXP3, IKZF2). The Tregs FOXP3 cells highly expressed inhibitory genes, including TIGIT, CTLA4, and ENTPD1, and they also relatively expressed high levels of costimulatory molecules CD28, TNFRSF14, ICOS, TNFRSF9, which stimulate the inhibitory activities (**Figure 5F**). The tumor-suppressive microenvironment mediated by Tregs is a significant obstacle to successful immunotherapy, suggesting that depletion of Treg cells, like immune checkpoint blockade of CTLA-4 or PD1/PDL1, could be a potentially effective immunotherapy for ovarian cancer (46).

Among CD8^+^ T cells, CD8 GZMK and CD8 GZMH T cells were characterized by relatively high cytotoxic genes granzyme K (GZMK) and granzyme H (GZMH), respectively. Meanwhile, these cells also positively expressed T cell exhaustion markers, including LAG3 and PDCD1, indicating that the CD8^+^ T cells are exhausted after initial activation in ovarian cancer (**Figure 5F**). In addition, we inferred the gene regulatory networks across the TILs subtypes by SCENIC method (**Figure 5G**). The regulon of PRDM1 was upregulated in CD8 GZMK and CD8 GZMH T cells, which is connected with terminal T cell differentiation and contributes to the maintenance of an early memory phenotype and cytokine poly-functionality in TILs after knockout (47). Consequently, we concluded that PRDM1 might be one of the factors contributing to the exhaustion of CD8 GZMK and CD8 GZMH T cells. Indeed, verified by bulk RNA-seq from the TCGA dataset, it was a slightly significant Spearman correlation between PRDM1 expression and immune exhausted infiltrate in ovarian cancer (Cor:0.41, FDR: 2e^−12^, **Figure S10C**). Beyond that, higher expression of PRDM1 predicted a worse prognosis in ovarian cancer (**Figure 5H**).

Next, we performed pseudo-time trajectory analysis to explore the dynamic states and cell transitions of CD4^+^ IL7R to CD8^+^ T cells *via* Monocle2. In the developmental trajectory, CD4^+^ IL7R started as a root, and gradually evolved into CD8 GZMK and CD8 GZMH, presenting a binary branched structure in which one side was the end of exhausted T cells, and the other side was the end of cytotoxic T cells (**Figure 5E**). In HG_M, the proportion of CD8^+^ cytotoxic T cells was higher than in HG_P (**Figure 5I**). On the contrary, the percentage of exhausted CD8^+^ T cells in HG_P was more than that in HG_M (**Figure S10D**).

We noticed three sub-clusters expressing the NK cells marker: TYROBP, GNLY, and NKG7. NK CD56 cells were characterized by NCAM1 (CD56), NK IL7R cells were characterized by IL7R, and NKT cells were identified by the specific T-cell markers including CD3D and CD8A (**Figures 5C, D**). NKT cells strongly expressed the GZMB, GZMA, GZMH, and PRF1 genes, indicating that they promoted tumor cytotoxicity in ovarian cancer (**Figure 5F**). Generally speaking, NK CD56 and NKT cells were more enriched in HG_P and HG_M than EC_P (**Figure S10E**).

### Trajectory Reconstruction of HGSOCs Revealed Monocyte-to-Macrophage Differentiation

**Figure 6:**
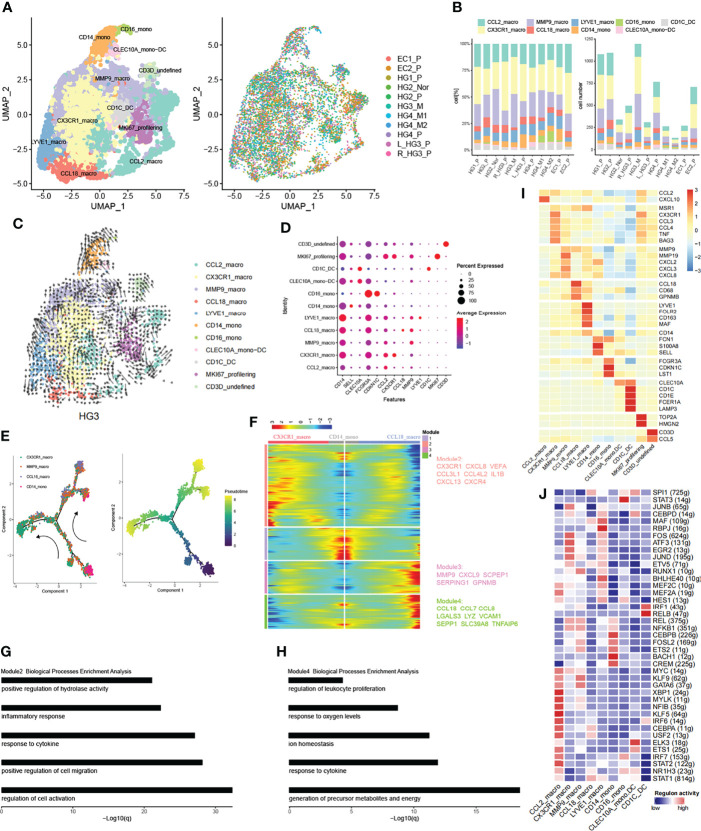
Subpopulations of myeloid cells in HG_M. **(A)** The UMAP projection of 7265 myeloid cells of 11 samples from six patients (indicated by labels and colors). **(B)** Proportion and cell number of each myeloid subtype in 11 samples. **(C)** RNA velocity of each myeloid subtype. **(D)** The dot plot displays canonical cell markers. **(E)** Trajectory reconstruction of monocyte evolved into macrophages. **(F)** Dynamics gene expression profile during monocyte-to-macrophage terminal differentiation. **(G**, **H)** Biological processes enrichment analysis of module 2 and module 4. **(I)** Heatmap of significant genes in each subtype. **(J)** Activate regulons in each myeloid subtype.

Tumor-infiltrating myeloid cells (TIMs) are critical regulators in tumor progression, playing essential roles in modulating tumor inflammation and angiogenesis (48, 49). Altogether, 7265 myeloid cells were collected, revealing 23 subsets through the ROGUE statistic (Version 1.0) (50) purified the cell population (**Figures S11A, 11B**). Then, we identified four common linages (cDCs, monocytes, or macrophage and monocytes derived DC) based on canonical cell markers and they were well presented in each sample (**Figures 6A, B, D**, **S11D, 11E**). Moreover, a subset of myeloid cells expressed myeloid/T-cells markers simultaneously (CD3D_undefined), which was not discussed below.

Monocytes are the progenitors of monocytes-derived macrophages and contribute to the overall coordination of immunity (51). Correspondingly, Monocytes can be separated from macrophages based on phylogenetic reconstruction (**Figure S11F**). CD14_mono cells were characterized by CD14, SELL, and S100A8/9, representing classical monocytes and being recruited during inflammation (**Figure 6I**). They also highly expressed FCN1, a complement system protein that defends against infectious agents (52). CD16_mono cells were less abundant and represented non-classical monocytes with high expression of FCGR3A (CD16), CDKN1C, LST1, and low expression of CD14. Similarly, CD16_mono cells expressed FCN1 at high levels but were more enriched in HG_M (**Figure S11C**).

DC cells were classified according to their origin and typical genes. CLEC10A_mono-DC with lower abundance was characterized by CLEC10A and CD14 representing monocyte-derived dendritic cells. CD1C_DC cells represented a classic cDCs subset, with high expression of CD1C, CD1E, and CLEC10A.

Macrophage cells were characterized by tissue-resident and their pro-inflammatory or anti-inflammatory function. CCL2_macro represented early-stage macrophage with the expression of CCL2. CX3CR1_macro expressed genes involved in immune modulation of chemokines, such as CCL3, CCL4, and CXCL8. What’s more, CX3CR1_macro significantly secreted BAG3 (**Figure 6I**), a multifunctional protein, which can combine with a specific receptor IFITM2 to induce the release of factors that sustain the growth and metastasis of tumor (53). MMP9_macro expressed genes related to inflammatory chemokines (CXCL2, CXCL3, CXCL8) and genes like MMPs (MMP19, MMP9), which play an important role in tumor tissue remodeling. CCL18_macro expressed both M1 marker (CD68) and M2 markers (CCL18, GPNMB). By the way, CCL18 played a key role in recruiting immunosuppressive myeloid cells (54). Finally, LYVE1_macro represented resident macrophages with a high level of LYVE1 and FOLR2.

Early works have supported that macrophages can either originate from monocyte cells or tissue-resident macrophages (55–57). Accordingly, we employed the RNA velocity to explore the ovarian cancer lineage trajectories and we found that a small number of CD14_mono evolved toward MMP9_macro, while LYVE1_macro evolved toward CCL18_macro as well as toward MMP9_macro (**Figure 6C**). To further investigate the dynamic change of genes during the differentiation of CD14_mono into macrophages in the ovarian lesions, we extracted classical monocytes (CD14_mono) and related macrophages (MMP9_macro, CCL18_macro, CX3CR1_macro) to delineate monocyte-to-macrophage differentiation by trajectory development analysis (**Figure 6E**). During the trajectory, CD14_mono were progenitor cells for MMP9_macrophage and then further separated into CX3CR1_macro and CCL18_macro. Profiling of gene expression dynamics along the trajectory had been divided into four modules (**Figure 6F**). Genes like CX3CR1, CXCL8, CXCR4, CCL3L1, VEGFA, and IL1B in Module 2 increased during the evolution of the branch of CX3CR1, whereas, they decreased during the evolution of the branch of CCL18_macro. Vice versa, Genes in Module 4 like CCL18, CCL7, and CCL8 were increased in the branch of CCL18_macro and reduced in the branch of CX3CR1. To reveal the biological characteristics of these two branches, we performed gene functional enrichment analysis. In the branch of CX3CR1_macro, the corresponding high expressed genes tend to be associated with inflammatory response and positive regulation of cell migration (**Figure 6G**), which is consistent with the suggestion that the high VEGF, CXCL8^+^ IL1β^+^ TAMs with the features of inflammatory could promote tumor progression in ovarian cancer (54, 58). Parallel results, in the branch which evolved into CCL18_macro, their corresponding high expressed genes were associated with metabolic precursor and energy production (**Figure 6H**).

We further characterized the functions of nine subtypes explained above by comparing pathway activities (**Figure S11G**). Pathways involved in angiogenesis, EMT, TNFA signaling *via* NFKB, and hypoxia were upregulated in CX3CR1_macro, MMP9_macro, and CD14_mono. These results indicate the potential tumor-promoting feature of CX3CR1_macro derived from CD14_mono. Finally, we applied SCENIC to identify TFs underlying each phenotype (**Figure 6J**). Interestingly, some recruited macrophage phenotypes shared similar TFs expression patterns with monocytes and tissue-resident macrophages.

### Intercellular Communication Networks

**Figure 7:**
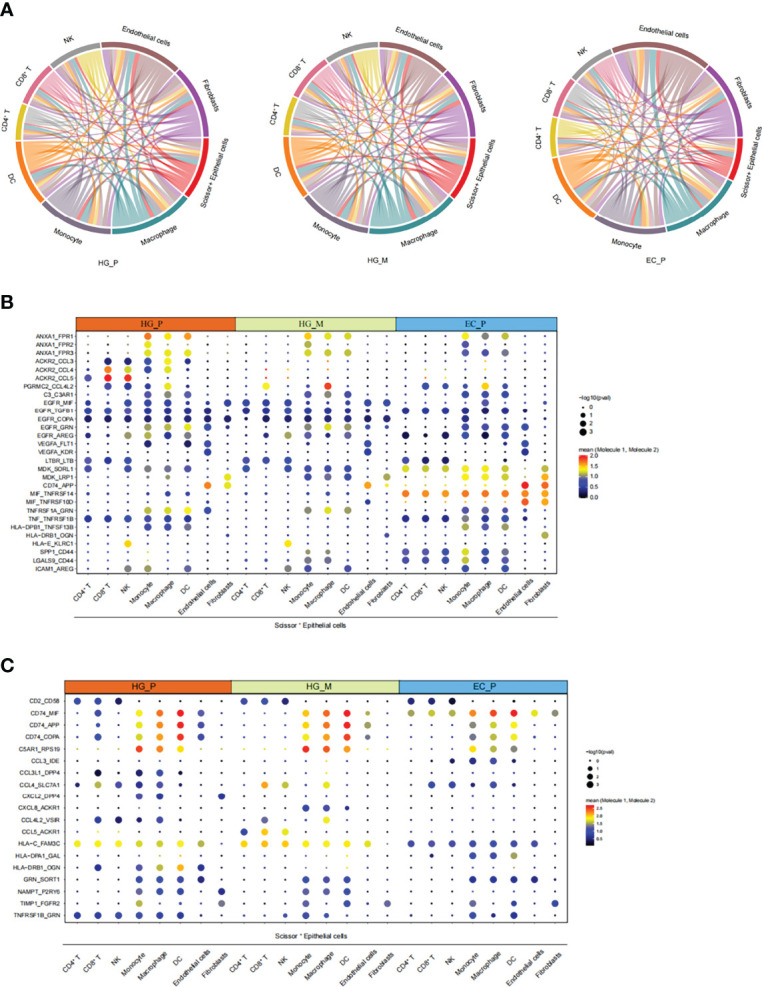
The intricate intercellular interplay in HG_P, HG_M, and EC_P. **(A)** Circos plot shows the intercellular interactions in HG_P, HG_M, and EC_P. Each line represents an interaction where one end represents a ligand that is expressed in one cell type and the other end represents a receptor that is expressed in another cell type. The thickness of each line corresponds to the number of distinct interacting pairs. **(B**, **C)** Dot plot shows the means of the average expression levels and the possibility of occurrence in selective interaction pairs.

As mentioned above, we have obtained cancer epithelial cells with poor prognosis (Scissor^+^ Epithelial cells), and we wondered what function they are involved in with the crosstalk of the tumor microenvironment in ovarian lesions. To this end, the CellphoneDB repository (Version 2.1.4) (59) was used to predict putative intercellular interactions between Scissor^+^ Epithelial cells and other cell types based on ligand-receptor signaling. Interestingly, many significantly overexpressed molecular pairs were associated with immunosuppression and HG_M showed a similar pattern to HG_P (**Figures 7B, C**). Compared with EC_P, Scissor^+^ Epithelial cells in HG_M and HG_P had more outgoing interactions with other cell types. Of note, macrophage and fibroblasts connected with Scissor^+^ Epithelial more frequently in HG_M (**Figure 7A**). When Scissor^+^ Epithelial cells expressed a relatively high level of EGFR as receptors, their corresponding ligands, such as AREG, COPA, GRN, MIF, and TGFB1, were widely expressed in other cells. When Scissor^+^ Epithelial cells expressed genes related to angiogenesis (VEGFA), the interactions (VEGFA_FLT1, VEGFA_KDR) were slightly abundant in HG_M. In addition, when Scissor^+^ Epithelial expressed ACKR2 acting as a receptor for chemokines including CCL3/CCL4/CCL5, their interactions were more abundant in HG_P than that of the other two groups (**Figure 7B**). It has been reported that ACKR2 is a scavenger receptor for chemokines and its deficiency against metastasis (60). And, CCL5 is important for the recruitment and activation of lymphocytes (61), so we proposed that ACKR2_CCL5 may weaken the recruitment and activation of lymphocytes, contributing to the metastasis of primary high-grade serous carcinoma. Among the three groups, the MDK_LRP1 molecule pair between Scissor^+^ Epithelial cells and myeloid cells expressed significantly, while their mean expression level in EC_P was higher than HG_P and HG_M (**Figure 7B**). MDK can combine with its receptor LRP1, which is beneficial to tumor-infiltrating macrophages, promoting myeloid inhibitory cell differentiation (MDSCs) (62, 63). Thus, MDK targeted therapy should suggest an effective treatment for ovarian cancer.

In general, these results revealed that the crosstalk between Scissor^+^ Epithelial cell and other cell types *via* diverse receptor-ligand signals may profoundly affect ovarian cancer development and metastasis.

## Discussion

Our current study has comprehensively characterized the dynamic variation of gene profiles during tumor progression in HGSOCs, as well as the heterogeneity of tumor cells, fibroblast cells, and immunophenotype, and the intricate intercellular interactions across HG_P, HG_M, and EC_P. We have identified unique subpopulations such as Scissor^+^ Epithelial cells, CAFs–Fibro_2, CX3CR1/CCL18 macrophages, and GZMK/GZMH CD8^+^ T cells. Those results provide a new perspective on the tumor microenvironment of ovarian cancer.

Epithelial cells were the largest cluster of cells, composing ~31% of the cells analyzed. However, current single-cell studies of ovarian cancer cannot explicitly distinguish cells with specific clinical phenotypes (24). To this end, Scissor^+^ cells with poor prognosis were identified by the Scissor algorithm. Consistent with the previous conclusion that the EMT signature is a potential factor for tumor invasion and metastasis (26, 27), the genes related to the EMT signature, including MGP, GAS1, and JUN, were found in Scissor^+^ cells of metastatic HGSOCs lesions. Moreover, during HGSOCs progression, several signaling pathways such as the cell cycle, tumor cell proliferation, oxidative phosphorylation, and TCA cycle, which needed energy metabolism, were markedly enhanced, suggesting that poly (ADP-ribose) polymerase (PARP) inhibition may be a targeted strategy for the treatment of metastatic HGSOCs (31–33). Strikingly, in our data, regardless of whether the primary tumor was from the left ovary or the right, the pathway activities generated by the tumor progression were basically consistent, as well as their copy number alterations in one segment of chromosomes.

Since the fibroblasts were the second-largest cluster cells analyzed, we observed Fibro_2, a subtype of CAF expressing the EMT program was specifically enriched in HG_M. This result supports that CAFs contribute to the EMT in HG_M and subsequently promote metastasis (64). Beyond that, the IL6_JAK_STAT3 signal pathway was also more enriched in Fibro_2. Consistently, JAK/STAT inhibitor JSI-124 has been proven to have an anti-tumor property in HGSOC cell lines (11). Combination therapies with the EMT or JAK/STAT inhibitor may help in the treatment of HGSOCs.

T cells are the crucial players in cancer immunotherapy (44, 45). Olalekan has revealed that ovarian cancer with high infiltration of CD8^+^ TOX^+^ and CD4^+^ GNLY T cells may be a good indication for patients (10). In our data, we found low T cell infiltration in EC_P compared with HG_P and HG_M. Furthermore, GZMH CD8 cells and GZMK CD8 cells simultaneously presented cytotoxicity and cell exhaustion. Notably, HG_M showed the highest cytotoxic of CD8^+^ T cells while the highest exhaustion of CD8^+^ T cells was in HG_P. Through the gene regulatory networks analysis, we speculated that PRDM1 may be involved in CD8^+^ T cells exhaustion and predicted that its high expression in ovarian cancer was associated with poor prognosis.

It has been manifested that TAM can promote the formation of niches before metastasis by secreting specific cytokines (65). They also can regulate the Tregs and Th17 cells to create immunosuppression, thereby promoting invasion and metastasis of ovarian cancer (66). On the basis of these theories, we have identified a subtype of TAM (CX3CR1_macro) with abundant production of BAG3, which can combine with IFITM2, leading to tumor metastasis (53). This TAM subtype also owns a high level of VEGFA, CXCL8, and IL1β, similar to previously reported TAM induced from monocyte with factor-1a stabilization in solid ovarian cancer that promoted tumor inflammation and metastasis (54).

By investigating the signaling network of Scissor^+^ Epithelial cells - other cells communication, we detected several receptor-ligand complexes that should be paramount for ovarian cancer development. Compared with HG_P and EC_P, stronger angiogenesis and tumor cell proliferation of intercellular interactions in HG_M. Metastasis of high-grade serous carcinoma may be related to the interaction between high expression of ACKR2 chemokine receptor and cytokines such as CCL5. The apparent pair of MDK_LRP1 among the three groups suggested that inhibition of the MDK_LRP1 pair might be an effective therapeutic target for ovarian cancer to reduce myeloid inhibitory cell differentiation (MDSCs).

We note that there are several limitations to our study. First, the number of patients with metastatic HGSOCs is small. Second, the clonal relationship of T cells was not investigated while T cell receptor therapy is an alternate therapy with great potential for ovarian cancer treatment (67). Hence, enlarging the cohort of metastatic HGSOCs and conducting immune profiling of T cell receptors and spatial transcriptomic may help unravel molecular mechanisms and elucidate the roles of different immune cells in HG_M.

In conclusion, our data have shed light on the tumor microenvironment of metastatic high-grade serous ovarian cancer at the single-cell level. Several novel markers and the potential therapeutic target detected in this study could provide valuable guidance for future clinical treatment.

## Materials and Methods

### Patients and Samples Collection

In this study, a total of eight samples, including one metastatic HGSOC matched two primary HGSOCs, one pair of matched primary HGSOC and normal ovary, one primary HGSOC and two ECs were collected from The Third Affiliated Hospital of Guangzhou Medical University, which have been subjected to pathological diagnosis. The clinical information of these patients is summarized in **Table S1**.

### Preparation of Single-Cell Suspension

Specimens collected from patients with ovarian cancer were minced into fragments (< 1 mm3) and digested with 0.25% trypsin-EDTA (GIBCO) and DNase I (Roche Diagnostics) for 30 min at 37°C with agitation. The dissociated cell suspension was filtered through 70 μm strainer (BD Falcon), washed with cold PBS, and centrifuged at 4°C, x400g for 5 min. The cell pellet was resuspended in serum-free DMEM for further use.

### Droplet-Based scRNA-Seq Data Preprocessing

The Cell Ranger (Version 6.0.2) pipeline generated raw gene expression matrices with human reference genome GRCh38. After removing doublets with DoubletFinder (Version 2.0.3) in each sample individually, the remaining cells were imported into R software (Version 4.1.0) for subsequent analysis by the Seurat package (Version 3.2.3). Cells with > 200 genes detected, genes expressed >5 cells, and genes expression >0 in all cells were selected for further analysis. Low-quality cells were removed according to the following criteria: unique molecular identifiers (UMIs) <500; genes <250 or genes >11000; UMIs derived from the mitochondrial genome >25%. After quality control, the gene expression was normalized by NormalizeData function, and cellular sequencing depth was adjusted by the SCTransform method.

### Multiple Datasets Integration

To integrate multiple datasets across three conditions, we used the integration methods described at https://satijalab.org/seurat/v3.0/integration.html and https://hbctraining.github.io/scRNA-seq/lessons/06_SC_SCT_and_integration.html. The Seurat package (version 3.0.0) was used to assemble multiple distinct scRNA-seq datasets into an integrated and unbatched dataset. In brief, we used Sctransform to regress out confounding factors: number of UMIs, percentage of mitochondrial RNA, estimating the variance of the raw filtered data, and identifying the 3000 most variable genes. After that, we performed canonical correlation analysis (CCA) and then “integrated” the conditions to overlay cells that were similar or had a “common set of biological features” between groups.

### Identification of Major Cell Types and Their Phenotype

Differential gene expression analysis was performed for clusters generated at various resolutions by both the Wilcoxon rank-sum test of Single-cell Transcriptomics (MAST) using the FindMarkers function. Annotation of the resulting clusters to cell types was based on the expression of marker genes along with the cell types assigned by SingleR packages (version ‘1.6.1’).

### TCGA Subtype Classification

Single cell subtypes were classified by the consensusOV (version 1.14.0) package, with get.consensus.subtypes function, which also returns random forest probabilities for each subtype. The core principle of consensusOV is that it: 1. standardizes genes in each dataset to the same mean and variance, 2. computes binary gene pairs based on the standardized expression values.

### CNV Estimation and Identification of Malignant Cells

We used an approach described previously to infer CNVs from the scRNA-seq data. Its R code was provided online. (https://github.com/broadinstitute/inferCNV) We set the cut off=0.1, denoise=TRUE, HSMM=TRUE, and hclust_method=‘ward.D2’. Immune cells from normal samples were considered as putative nonmalignant cells as control, and their CNV estimates were used to define a baseline. All epithelial cells from the ovarian tumor sample were used as input.

### Distinguish Phenotype-Associated Cells

To link cells with specific phenotypes, we used the Scissor algorithm, a novel R package (Version 2.0.0) to identify the populations of the single-cell data associated with given phenotypes. (https://github.com/sunduanchen/Scissor)

Scissor integrates phenotype-associated bulk expression data and single-cell data by quantifying the similarity between every single cell and bulk sample. To identify relevant subpopulations, it then optimizes a regression model on the correlation matrix with the sample phenotype. The core formula of Scissor is as follows:


$$\frac{\text{min}}{\beta }-\frac{1}{n}l\left(\beta \right)+\lambda \left\{\alpha {∥\beta ∥}_{1}+\frac{1-\alpha }{2}\beta^{T}L\beta \right\}$$

where L is a symmetric normalized Laplacian matrix; the tuning parameter λ controls the overall strength of the penalty, and α balances the amount of regularization between smoothing and sparsity. The phenotype-related cell subsets of interest are selected using the non-zero coefficient β solved for by the optimization model described above.

Statistical test: The scissors algorithm incorporates a reliability test to rule out false associations between identified cell subsets and bulk phenotypes. This statistical test can determine whether the inferred phenotype-cell association is reliable (P<0.05) or a false positive. First, it performs k-fold cross-validation (CV) on the correlation matrix S and estimates the cell coefficients in Scissor using only the training set. The predictive performance of the trained Scissor model is evaluated on the test set, and an averaged evaluation metric is obtained as the actual test statistic. Second, the bulk sample labels are randomized multiple times to break the original bulk phenotype-genotype relationship. Predictive performance at the random level was quantified by performing the same Scissor analysis and CV assessment using each permuted batch of data, obtaining the background distribution of the corresponding assessment measures. Finally, the actual test statistic calculated in the raw data is compared to the background distribution value. The reliability significance test p-value is the number of permutations-based test statistics above (or below) the actual test statistic divided by the number of permutations. In this study, the evaluation measures used in reliability significance tests were mean squared error (MSE) for linear regression (smaller is better), area under the ROC curve (AUC) for classification (higher is better), and agreement Sex index (C-index) for Cox regression (higher is better).

In actual operation, we set the family=“ cox”, alpha=0.077 for the HG group, and alpha=0.0265 for the EC group to select the phenotype-associated cell subpopulations by a Cox regression model. The number of the Scissor selected cells should not exceed 20% of the total cells in the single-cell data.

### SCENIC Analysis

The regulons and TF activity (AUC) for each cell were calculated with the SCENIC (version 1.2.4) pipeline with motif collection version mc9nr, using per cell type with raw count matrices as input. We used GRNBoost (in Python) instead of GENIE3 to detect positive and negative associations for a bigger dataset. The function of exportsForGRNBoost was used to generate a gene expression matrix and TF list in special formats for GRNBoost to load.

### Trajectory Inference Analysis

Trajectory analysis was performed using Monocle 2 (version 2.20.0), We assessed the significant gene by the differential gene expression analysis, and DEGs between the clusters were applied for dimension reduction using the reduceDimension function. Genes that changed along with the pseudotime were calculated and visualized with the plot_pseudotime_heatmap and the genes were clustered into subgroups according to the gene expression patterns. To identify the genes that separate cells into branches, the branch expression analysis modeling (BEAM) analysis was performed and genes resulting from the BEAM analysis with a q-value < 10−4 were separated into groups and visualized with the plot_genes_branched_heatmap function.

### Estimations of RNA Velocities by Velocyto Package

In order to smoothly assess spliced and un-spliced mRNAs, we needed to convert the bam file to loom file by the function of run10x, provided by velocyto.py. Next, we merged multiple loom files by the function of loompy.combinem and then loaded the merged loom file into R software to combine analysis with the Seurat package.

### Ordering the Gene Expression During Cell State Transitions

The genes, including the human surface proteins and transcription factors (TFs), act as on/off switches between cell states and are discovered by GenSwitches R packages (Version 0.1.0). The workflow of GeneSwitches is as follows:

1. Binarize gene expression data into 1(on) or 0(off).
2. Fit logistic regression on the binary states of gene expression and estimate switching time.
3. According to the default Settings, the poorly fitting genes are filtered and specific genes are extracted for plotting.

### Gene Set Variation Analysis (GSVA)

Pathway analyses were performed on the 50 hallmark pathways described in the molecular signature database. We also evaluated the activity of 65 specific KEGG pathway activities from the Canonical pathway KEGG subset. To assign pathway activity estimates to individual cells, we applied GSVA (Version ‘1.34.0’) with standard settings.

### Enrichment Analysis of Marker Genes

Gene Ontology (GO) enrichment and Kyoto Encyclopedia of Genes and Genomes (KEGG) pathway analysis of differential genes was implemented by the clusterProfiler (version 4.0.2) package. Reactome and Hallmark pathways analysis on differential genes was implemented by Metascape web-based portal (68).

### Cell Cycle Scoring Assign

Each cell in the epithelial subpopulation was assigned a cell cycle-related score based on the gene expression of the G2/M and S phases. The “CellCycleScoring” function of the Seurat package was used to calculate the cell cycle score and store G2/M and S phase scores into data objects to predict the cell state.

### TCGA Database

The cohort of TCGA ovarian cancer data was downloaded from https://xenabrowser.net/datapages/. The gene expression matrix and clinical phenotypes of HGSOCs and ECs were assessed using different datasets (GDC TCGA Ovarian Cancer (OV) & TCGA Endometrioid Cancer (UCEC)).

### Survival Analysis

The Kaplan-Meier method evaluated ovarian cancer survival utilizing the KM plotter database (https://kmplot.com/analysis/).

We set split patients by ‘Auto select best cutoff’, which chooses the best performing threshold as cut-off among all possibilities between the lower and upper quartiles. All datasets provided by the KM plotter were taken into consideration for analysis.

### Definition of Cytotoxicity and Exhaustion Scores

Cytotoxicity and exhaustion scores were calculated by the average expression of the genes from the predefined gene sets in CD8^+^ T cells of each group. To implement this method, the AddModuleScore function of the Seurat package was applied as previously described. We used eight cytotoxicity associated genes (GZMA, GZMB, GZMH, GZMH, IFNG, NKG7, PRF1, GNLY) and seven exhaustion associated genes (LAG3, TIGIT, PDCD1, HAVCR2, CTLA4, ENTPD1, BTLA) to define cytotoxicity and exhaustion scores

### Correlation Analysis Between Immune Infiltration and Single Gene Expression

Spearman correlation between mRNA expression and exhausted infiltrate in ovarian cancer was performed by immune cell abundance pattern of Gene Set Cancer Analysis (GSCA) (69).(http://bioinfo.life.hust.edu.cn/GSCA/#/).

### Statistics

Statistical analysis was carried out using R and Bioconductor.

### Cell-Cell Communication Network

To investigate potential interactions between different cell types in the ovarian cancer tumor microenvironment, cell-cell communication analysis was performed as described previously by the CellPhoneDB Python package (Version 2.1.4), a publicly available repository of curated receptors and ligands and their interactions. Prediction of enriched receptor-ligand pairs between two cell types was derived from the expression of a receptor by one cell type and the expression of the corresponding ligand by another cell type. By default, only ligands and receptors expressed in at least 10% of cells in a given cell cluster were taken into consideration.

Pairwise comparisons between selective cell types were performed. We randomly permuted the cluster labels of all cells 1000 times to determine the mean of average ligand and receptor expression levels of the interactions, generating a null distribution for each receptor-ligand pair. A p-value for the likelihood of cell-type specificity of the corresponding receptor-ligand complex was obtained by calculating the proportion of the means as high as or above the actual mean. Then, we can select biologically relevant interactions.

## Data Availability Statement

Single-cell RNA data reported in this paper have been deposited (PRJCA009148) in the Genome Sequence Archive in the BIG Data Center under accession number HRA002345 that are publicly accessible at https://ngdc.cncb.ac.cn/gsa-human/. All relevant data are available from the corresponding author upon reasonable request.

## Ethics Statement

The studies involving human participants were reviewed and approved by The Third Affiliated Hospital of Guangzhou Medical University (No: 2017-075). The patients/participants provided their written informed consent to participate in this study.

## Author Contributions

XS and DS directed and oversaw the project. YT, DZ, and TC collected ovarian tissue samples. YD performed comprehensive bioinformatics analyses. YD, YB, and DS wrote the manuscript with all the other authors participating in the discussion, data interpretation, and manuscript editing. All authors contributed to the article and approved the submitted version.

## Funding

This research was supported by the Natural Science Foundation of Guangdong Province (no. 2020A1515010082), National Natural Science Foundation of China (NSFC, Project 81773012), National Science Foundation of China (grant 82170242 and 81570454), Science and Technology Planning Project of Guangzhou (202102010003), The Project for Key Medicine Discipline Construction of Guangzhou Municipality (no.2021-2023-17), and China Postdoctoral Science Foundation (2019M662858).

## Conflict of Interest

The authors declare that the research was conducted in the absence of any commercial or financial relationships that could be construed as a potential conflict of interest.

## Publisher’s Note

All claims expressed in this article are solely those of the authors and do not necessarily represent those of their affiliated organizations, or those of the publisher, the editors and the reviewers. Any product that may be evaluated in this article, or claim that may be made by its manufacturer, is not guaranteed or endorsed by the publisher.
