# Supplementary material for: Single-Cell RNA-Sequencing Atlas Reveals the Tumor Microenvironment of Metastatic High-Grade Serous Ovarian Carcinoma
Source: Front Immunol. 2022 Jul 22;13:923194. doi: 10.3389/fimmu.2022.923194 (PMC9354882; doi:10.3389/fimmu.2022.923194)
Supplement: Supplementary file 1 [file DataSheet_1.docx]

Supplementary Material

# Supplementary Tables and Figures

## Supplementary Tables

Table S1: Clinical characteristics of the six patients

| Patient number | Age(years) | Stage | TNM classification | Pathological subtype |
| --- | --- | --- | --- | --- |
| HG1  HG2  HG3  HG4  EC1  EC2 | 64  65  47  70-75  49  55 | IIIC  IIC  IIC  IIIC  ⅢA  IC2 | pT2cNxMx  pT2cN0Mx  T2cNxMx  pT3cNxM0  pT3aNxMx  NA | High-grade serous carcinoma  High-grade serous carcinoma  High-grade serous carcinoma  High-grade serous carcinoma  Endometrioid carcinomas  Endometrioid carcinomas |

Table S2: Sequencing metrics of the samples included in this study.

| Samples | Cells | Sample type | Tumor site | Read | Saturation (%) | QC (cells) |
| --- | --- | --- | --- | --- | --- | --- |
| HG1_P  HG2_P  HG2_nor  R_HG3_P  HG3_M  L_HG3_P  HG4_P  HG4_M1  HG4_M2  EC1_P  EC2_P | 5598  8560  62333  7072  6376  8172  6351  1432  1501  6194  8973 | Tumor  Tumor  Normal  Tumor  Tumor  Tumor  Tumor  Tumor  Tumor  Tumor  Tumor | Ovarium  Ovarium  Ovarium  Ovarium  Peritoneum  Ovarium  Ovarium  Peritoneum  Peritoneum  Ovarium  Ovarium | 640,006,280  565,101,293  615,936,188  610,067,718  617,613,946  553,559,125  400,459,655  250,974,825  280,613,748  585,246,291  691,756,880 | 72.7%  72.7%  81.9%  65.8%  62.6%  68.8%  78.2%  93.9%  92.9%  81.6%  65.2% | 4966  5168  5784  6500  6388  7339  6012  1398  1441  5908  7205 |

Table S3: Functional enrichment analysis based on the upregulated genes in ECs Scissor^+^ cells versus All other cells (scissor^-^ and background cells).

| Pathological subtype | Category | Description | Log10(q) | Genes |
| --- | --- | --- | --- | --- |
| EC | Reactome Gene Sets | Metabolism of RNA | -13.68 | HDGF, RPS5, H3F3A, RPS12, RPL8, LSM5, CFL1, PRDX3, PAPOLA, RPL10A, CLNS1A, RPL7, RPS8, YBX1, PRDX6, RPL18A, RPS17, MRPL3, TUBA1C, LSM4, KPNA2, GSTP1, EIF5A, GLUL, SLBP, PRDX2, SNRPD1, NCL, NHP2, RPS2, RPLP0, ARPC5, NPM1, PFN1, SNRPE, RPL17, EIF4A1, H2AFZ, NUDT8, RAN, PPA1, HMGA1, UBE2S, PSAT1, TUBA1B |
| EC | Reactome Gene Sets | Cell Cycle | -6.96 | CBX3, H3F3A, MCM7, PPP1CA, TUBA1C, GSTP1, CKS1B, PRDX2, YWHAQ, NHP2, RPS2, PPIA, NPM1, CA2, H2AFZ, RAN, NME1, PCNA, UBE2S, SFN, TUBB, TUBA1B, TYMS, HMGB2, CCND1, STMN1 |
| EC | Reactome Gene Sets | Formation of tubulin folding intermediates by CCT/TriC | -5.33 | CCT8, H3F3A, VDAC1, NUCKS1, HNRNPAB, TUBA1C, KPNA2, GLUL, CCT5, SLC25A5, NHP2, GAPDH, HMGB1, PPIA, NPM1, PFN1, CCT3, RAN, PCNA, SFN, TUBB, TUBA1B |
| EC | Hallmark Gene Sets | MYC_TARGETS_V1 | -30.68 | CBX3, HDGF, RPS5, MCM7, VDAC1, PRDX3, CLNS1A, KPNA2, CCT5, YWHAQ, SNRPD1, ILF2, NHP2, RPS2, RPLP0, PPIA, NPM1, EIF4A1, H2AFZ, CCT3, RANBP1, RAN, NME1, DUT, PCNA, TYMS |
| EC | Hallmark Gene Sets | E2F_TARGETS | -15.52 | MCM7, UNG, KPNA2, SLBP, CKS1B, SNRPD1, NCL, UBE2T, H2AFZ, RANBP1, RAN, NME1, HMGA1, DUT, PCNA, UBE2S, TUBB, HMGN2, HMGB2, CCND1, STMN1 |
| EC | Hallmark Gene Sets | MTORC1_SIGNALING | -4.99 | UNG, CACYBP, GAPDH, PPIA, TPI1, ENO1, PPA1, PSAT1, STMN1 |

## Supplementary Figures


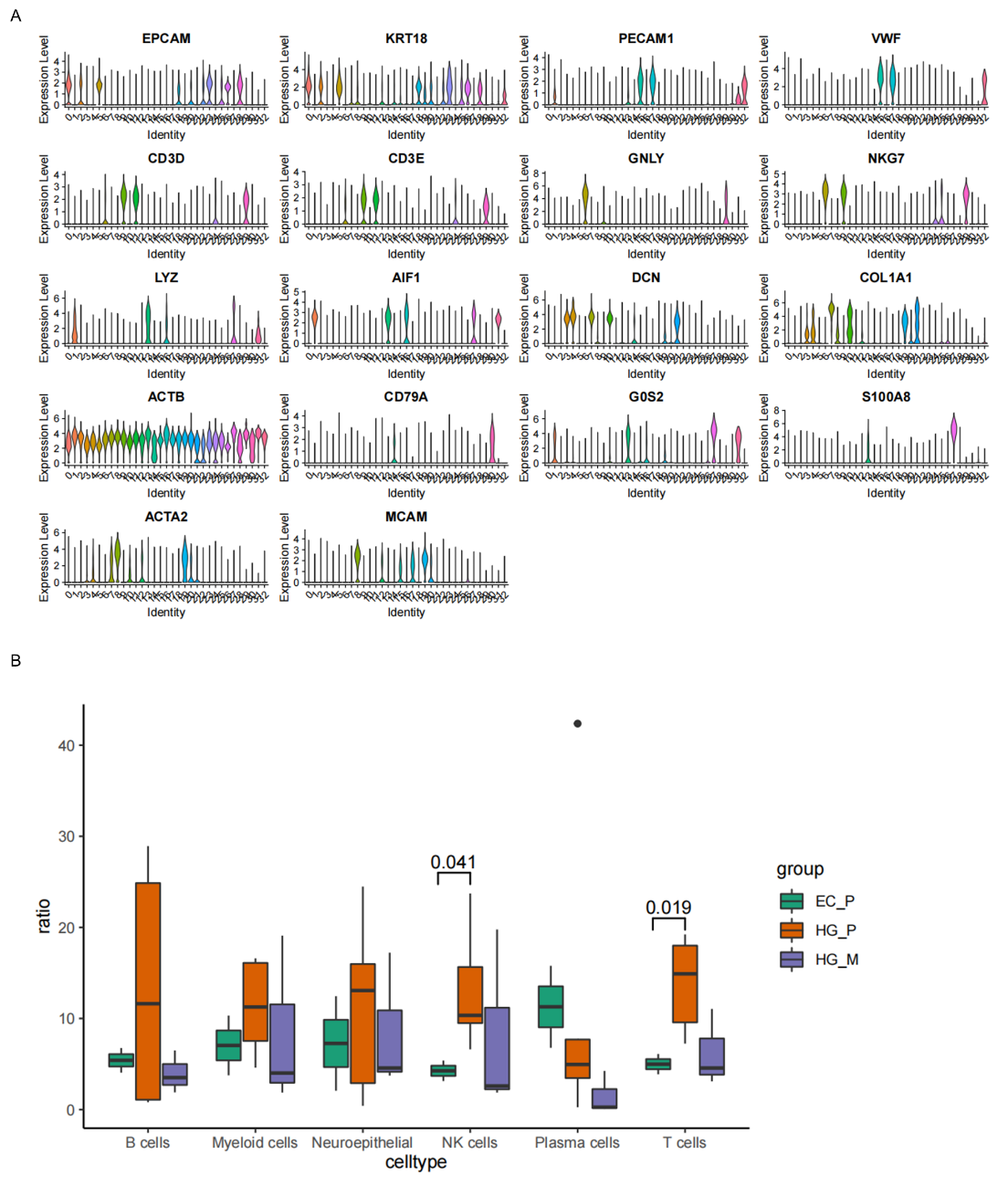


**Supplementary Figure1.** Expression of canonical biomarker expression and distribution of immune cells among HG_P, HG_M, and EC_P. (A) The violin plot shows the normalized expression of 14 marker genes for each of all clusters as displayed. (B) The box plot shows the proportion of each immune type (as indicated by the X-axis) in HG_P, HG_M, and EC_P. P-value was calculated by a two-sided unpaired T-test.


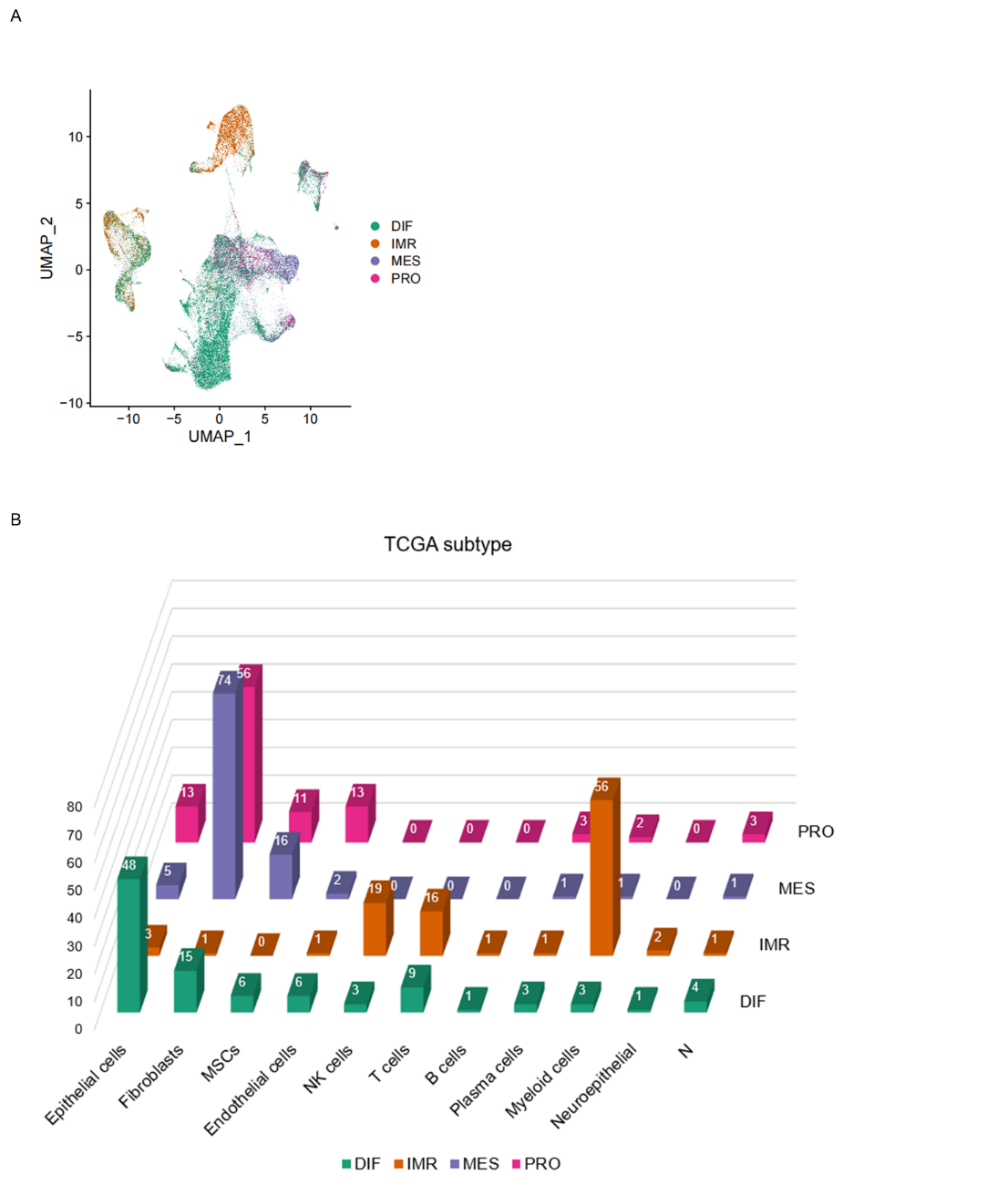


**Supplementary Figure 2.** Single cells assigned TCGA subtype. (A) The UMAP projection shows each cell subtype assignment. (B) The three-dimensional plot of subtype scores (labeled by white color) is based on the ratio of the number of certain molecular subtypes per cell in each subtype called by the consensusOV package to the total number of these subtypes.


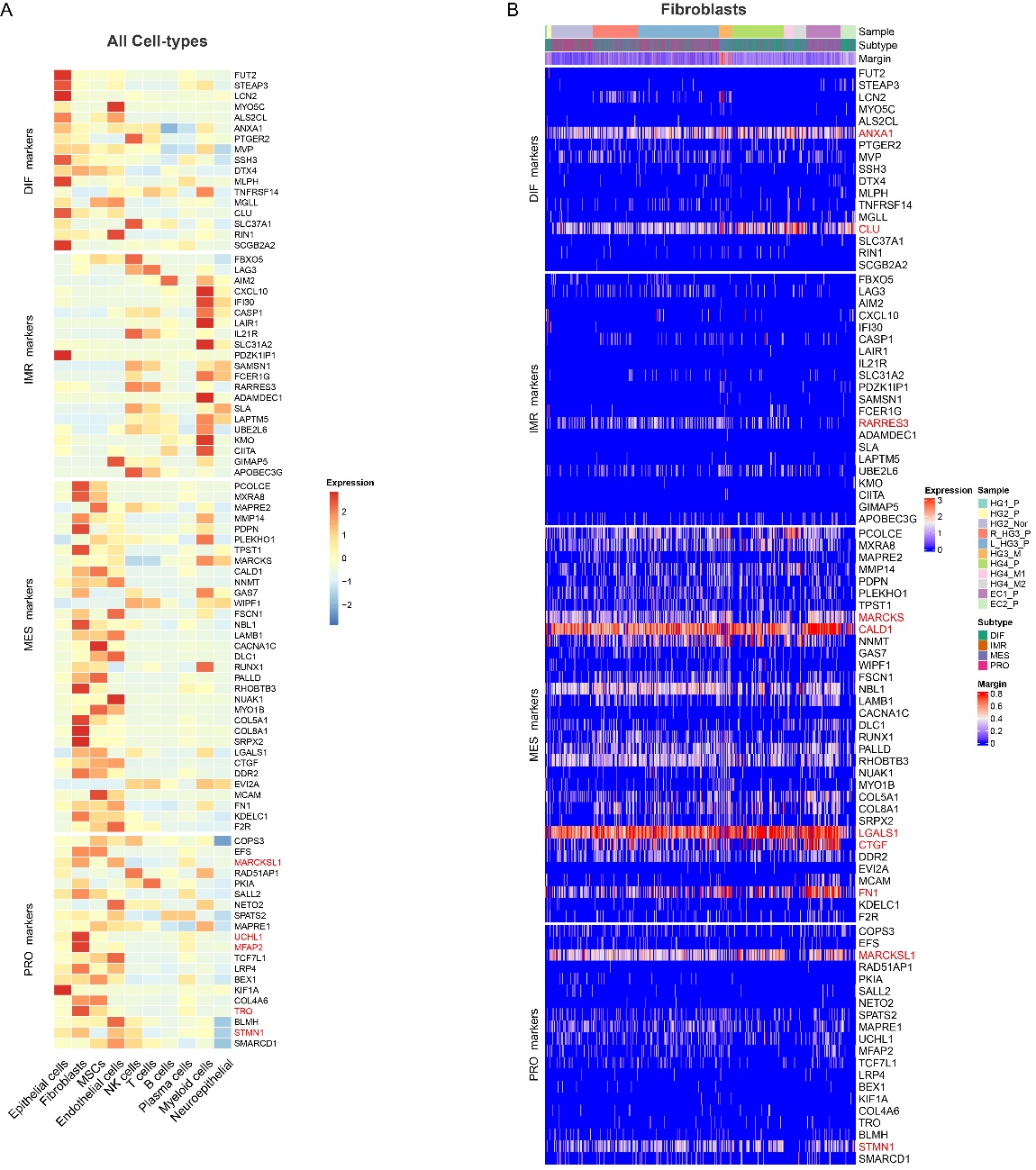


**Supplementary Figure 3.** Heatmap of genes provided by Verhaak for TCGA-subtype classified. (A) Heatmap shows the average expression of subtype-markers across all cell types. (B) Heatmap shows the expression of subtype-markers in each cell of fibroblasts (Margin: the difference between the top two subtype scores for each tumor, the high margin means high confidence of classification.).


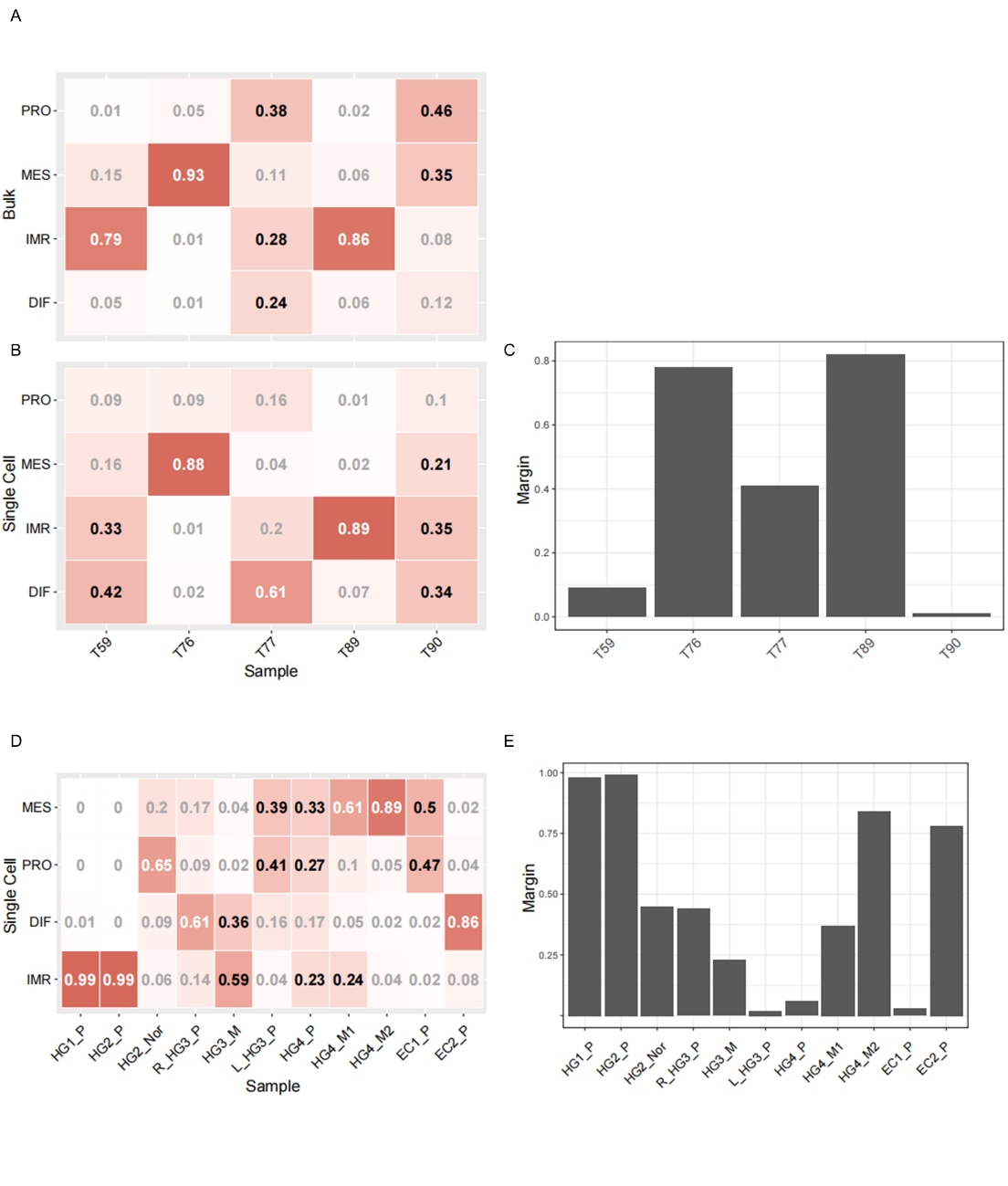


**Supplementary Figure 4.** TCGA-subtypes classification of pseudo-bulk tumors and bulk tumors. (A) Subtypes classification of bulk RNA-seq from public data. (B) Subtypes classification of pseudo-bulk tumors (converted from single-cell data which matched (A) data). (C) The barplot shows the margin scores of different pseudo-bulk tumors (Margin: the difference between the top two subtype scores for each tumor, the high margin means high confidence of classification). (D) Similar to (B). (E) Similar to (C).


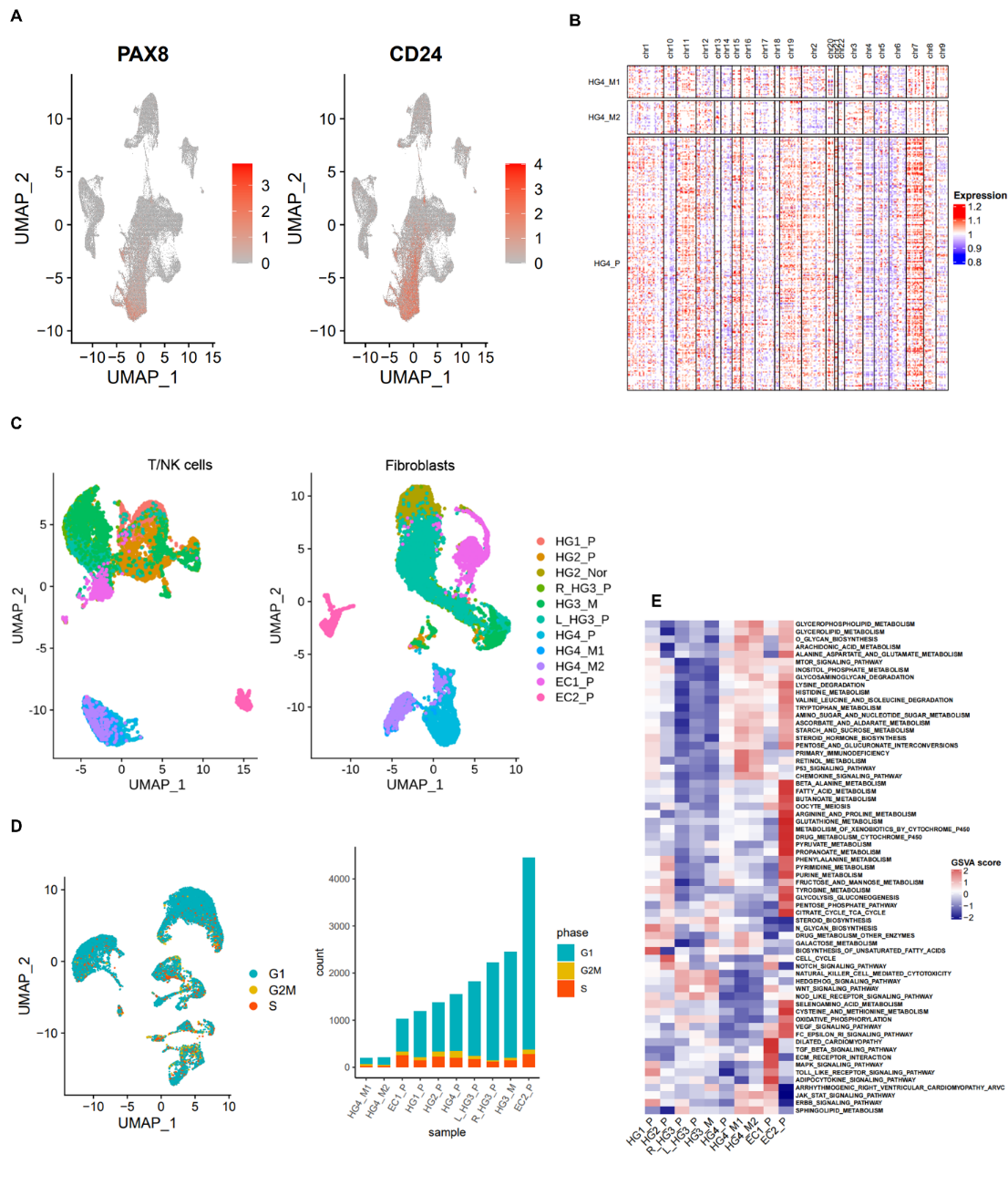


**Supplementary Figure 5.** Characterization of epithelial cells. (A) Expression of malignant marker genes (PAX8, CD24) in epithelial cancer. (B) The chromosomal landscape of copy number for primary tumors and metastatic tumors of HG4 patient. (C) The UMAP projection of T/NK cells and fibroblast cells from 10 tumors of 6 patients (indicated by labels and colors) reveals tumor-specific clusters. (D) The UMAP projection shows the difference of epithelial cells in phases of the cell cycle (left) and the distribution of the cell cycle phases in each tumor. (E) GSVA analysis of differential pathways is scored per cell among each tumor.


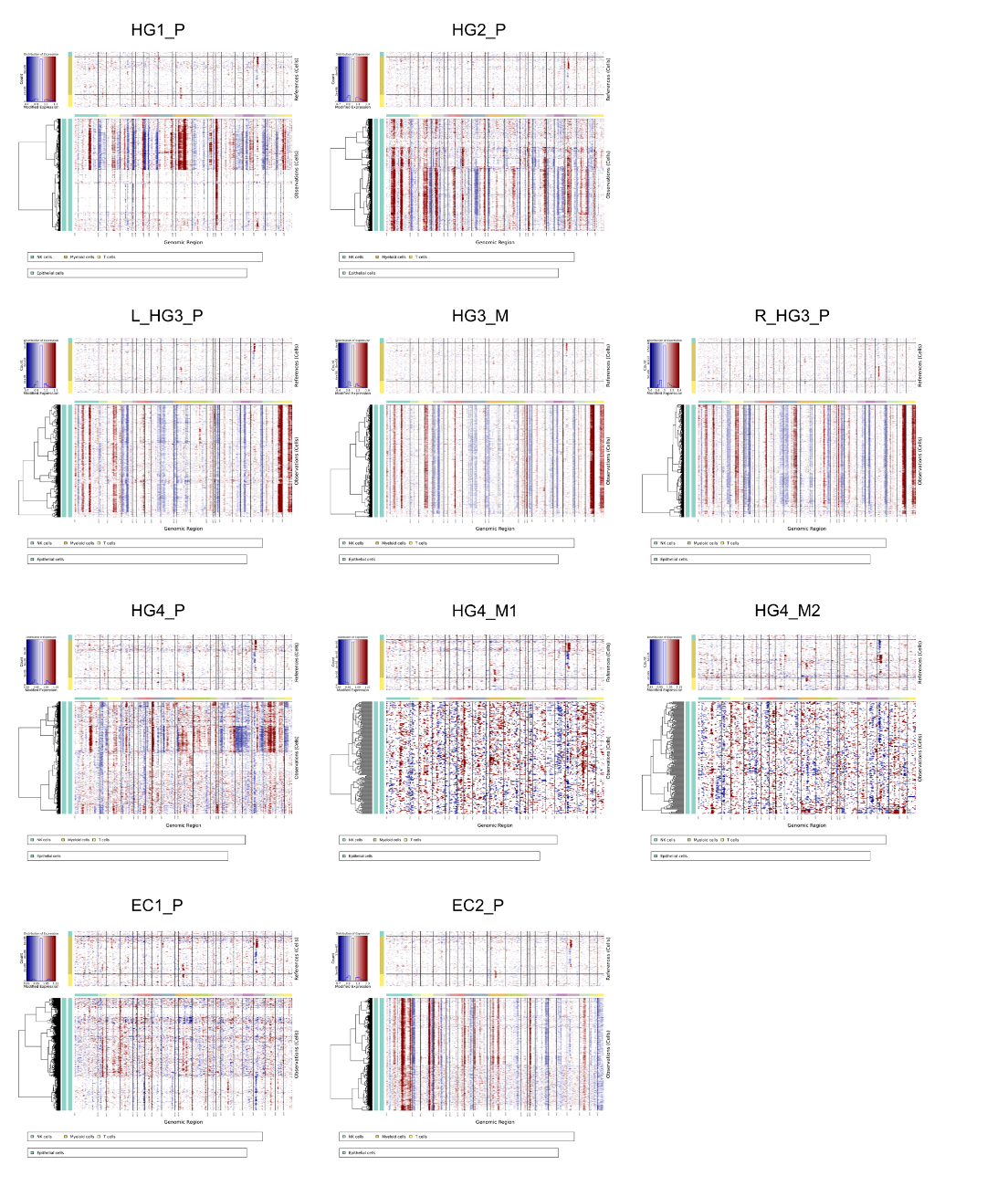


**Supplementary Figure 6.** Copy number profiles of each sample. Single cells copy number variation inferred from inferCNV. Normal references (extract from normal sample: HG_nor) are plotted at the top, where NK cells are green, myeloid cells are in brown, and T cells are in yellow. Tumor cells are plotted at the bottom, where red means amplifications and blue means deletions.


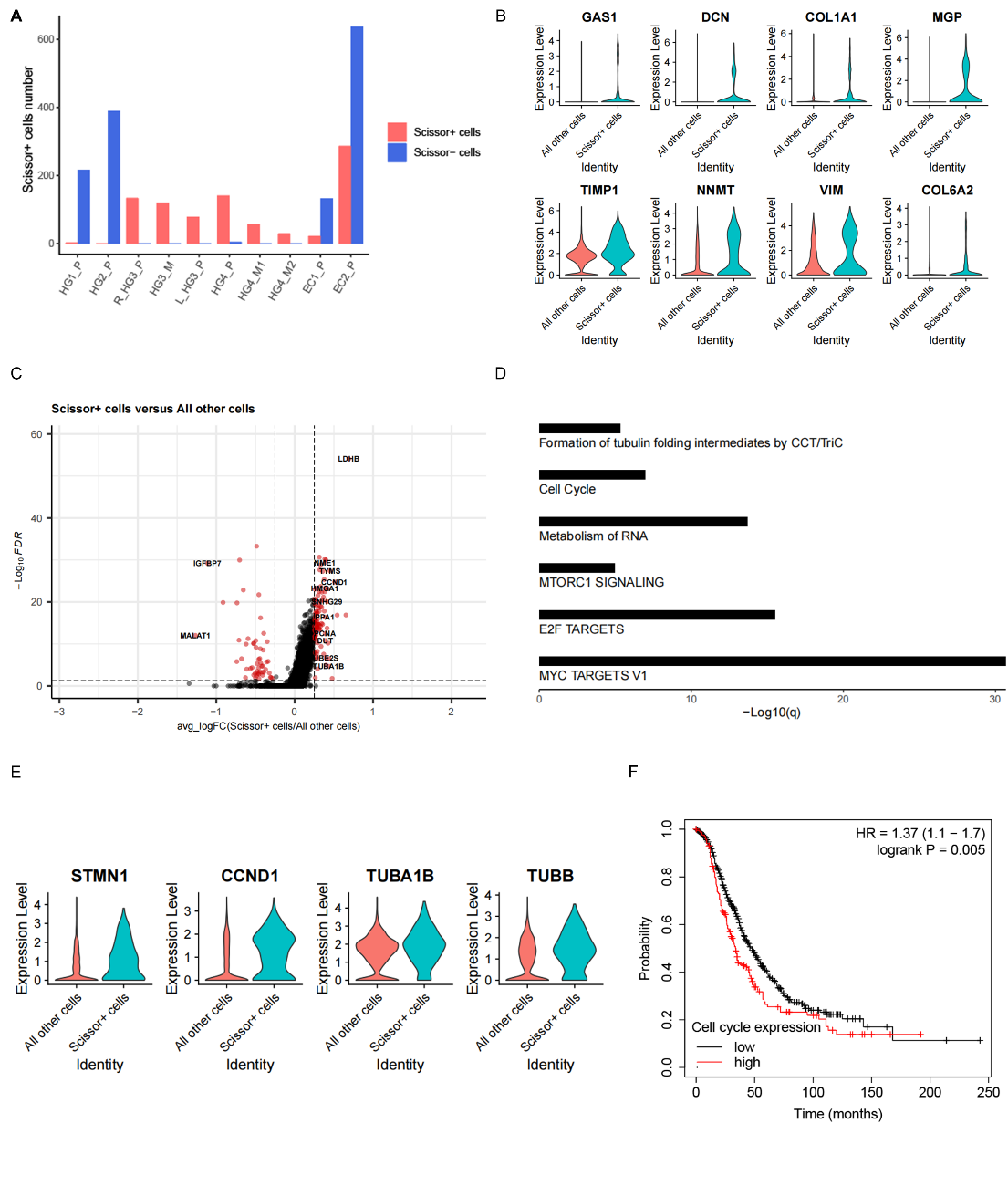


**Supplementary Figure 7.** Characterization of the Scissor^+^ cells in ovarian lesions. (A) The distribution of Scissor cells (Scissor^+^ cells mean worse prognosis; Scissor^-^ cells mean better prognosis) in each tumor. (B) The violin plot shows the expression of EMT-related genes between Scissor^+^ cells and all other cells (Scissor^-^ cells and Background cells) in HGSOCs. (C) Differentially expressed genes between Scissor^+^ cells and all other cells in ECs, each point represents a gene. Red: significant genes; Black: NS genes. avg_logFC: log 2 fold-change of the average expression between the two groups. ((log-FC > 0.25, FDR <0.05) (D) Enrichment of significant genes related to Reactome and Hallmark pathways. (E) The violin plot shows the expression of cell cycle-related genes between Scissor^+^ cells and all other cells in ECs. (F) Kaplan-Meier plot shows that high expression of cell cycle signature has shorter overall survival in ovarian cancer. The high and low patients are split by the mean expression of the EMT-related gene set.


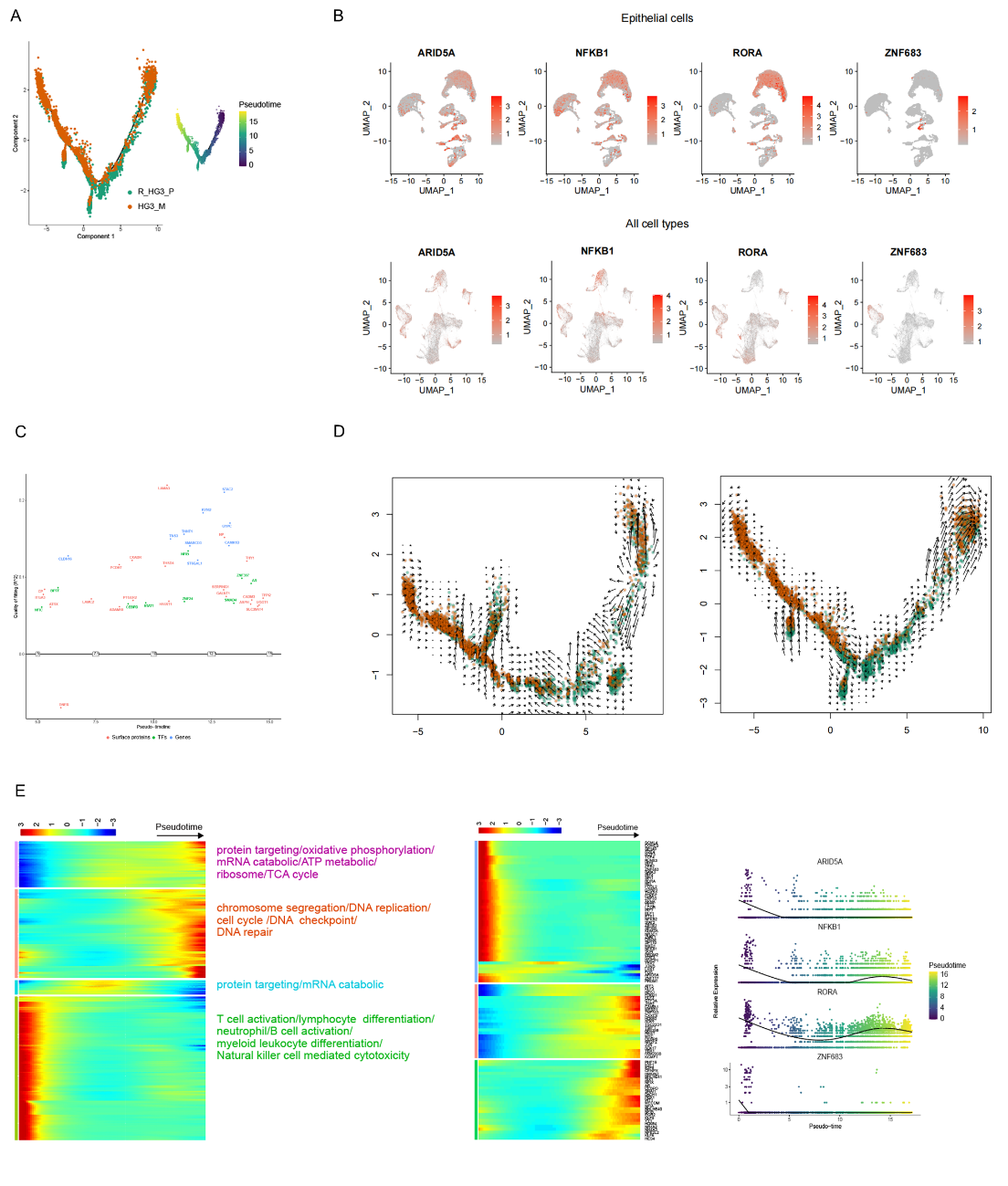


**Supplementary Figure 8.** Gene expression profiles in metastatic HGSOCs. (A) Monocle2 infers the development of epithelial cells along with pseudo-time (from patients HG3, R_HG3_P means the primary tumor from the right ovary). Pseudo-time legend from dark to bright indicates cancer progression from the early to late stage. (B) UMAP-plot displays immune-related TFs in the cluster of epithelial cells and other cell types. (C) Genswitches deduces the genes switch between cell states in R_HG3_P to HG3_M. (D) RNA velocity of metastatic HGSOCs along the trajectory. (left: L_HG3_P to HG3_M; right: R_HG3_P to HG3_M; green: primary, red: metastasis) (E) The heatmap displays the dynamic gene expression profiles during metastasis of ovarian cancer (left). The color key from blue to red indicates relative expression levels from low to light. The top annotated GO and KEGG terms in each cluster are shown. Differentially expressed transcription factor genes (TFs; middle) and the expression of specific TFs are on view along with the pseudo-time curve in (right).


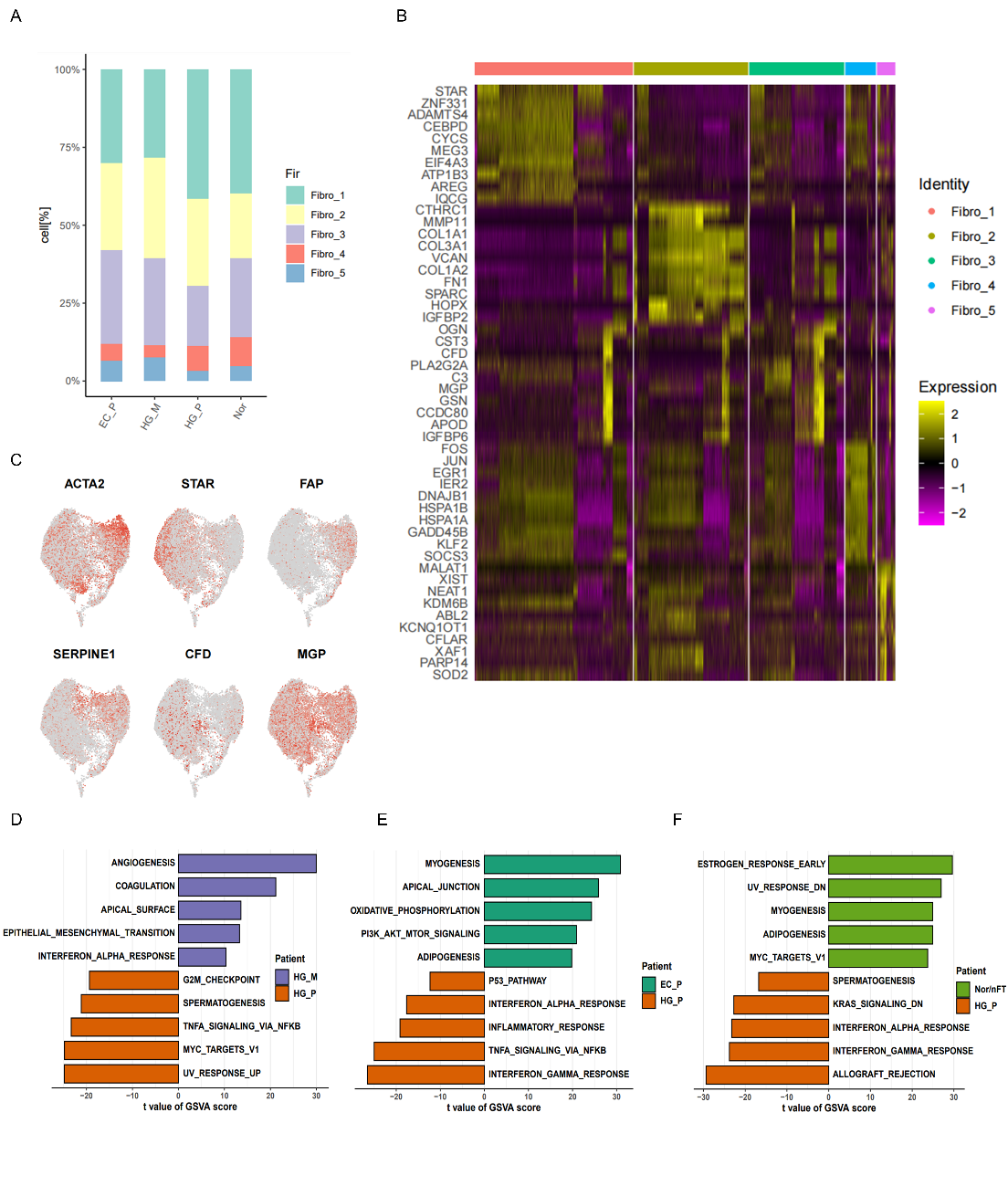


**Supplementary Figure 9.** Fibroblast subtypes in HG_P, HG_M, EC_P (A) The distribution of 5 fibroblast subtypes among EC_P, HG_P, HG_M. (B) The heatmap of top10 differentially genes across 5 fibroblast subtypes. The color key from purple to yellow indicates relative expression levels from low to light. (C) The UMAP shows the expression of the canonical biomarker. (D)(E)(F) Differentially expressed hallmark pathways of fibroblasts in HG_P compared with those in HG_M/EC_P/Nor/nFT as scored per cell by GSVA. The top 5 increased/decreased pathways are shown. T scores were obtained from a linear model by limma.


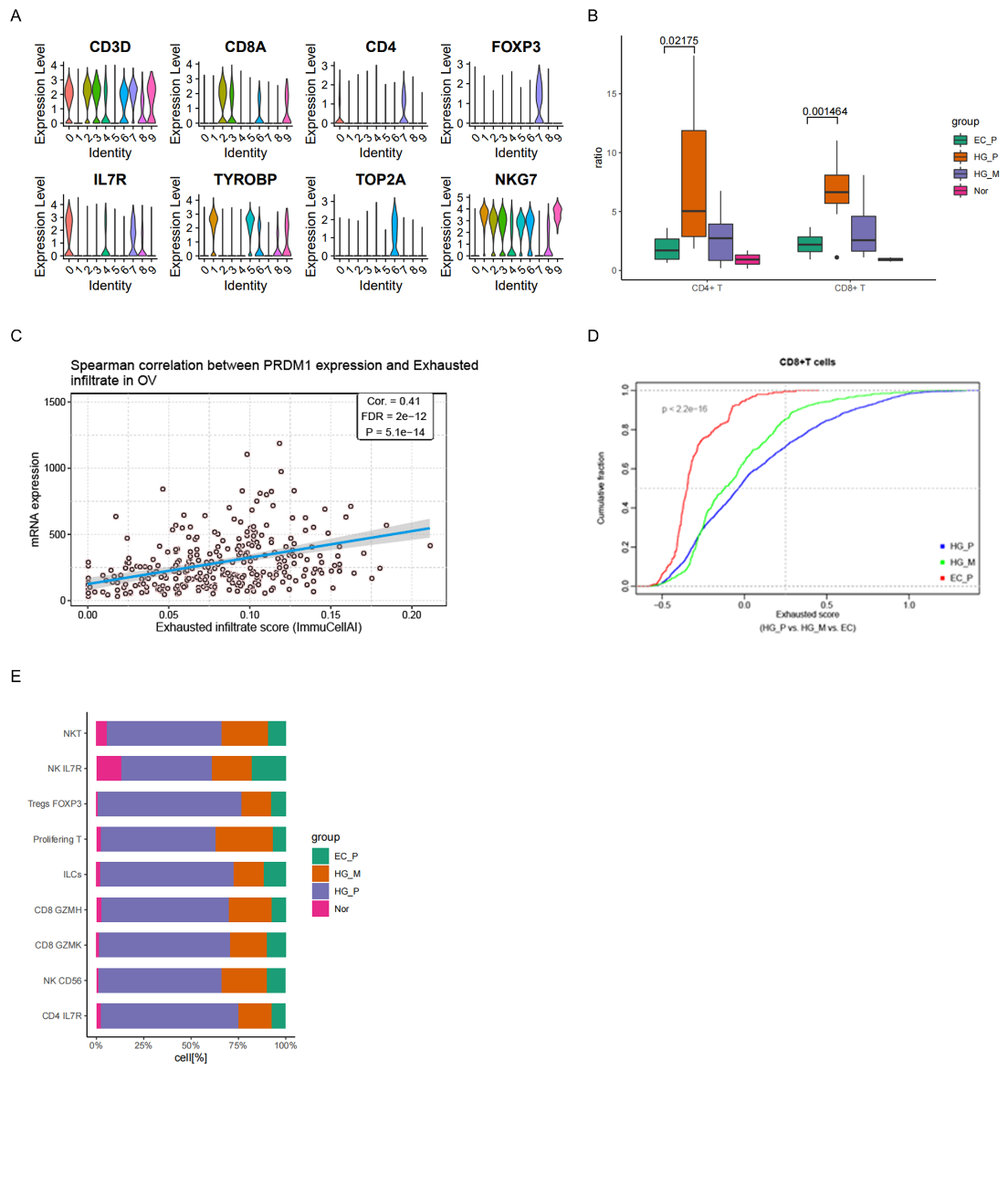


**Supplementary Figure 10.** Characterization of the TILs cells in ovarian lesions. (A) The violin plot shows the normalized expression of 10 marker genes for each of the 10 clusters as displayed. (B) The box plots show the ratio of CD4^+^ and CD8^+^ T cells in each group (as indicated by color). P value was calculated by a two-sided unpaired T-test. (C) Correlation analysis between PRDM1 expression and CD8^+^ T cell exhausted infiltrate using the immune cell abundance function of GSCA (Gene Set Cancer Analysis). (D) Cumulative distribution of exhausted CD8^+^ T cells between HG_P, HG_M, and EC_P. The cytotoxic score is calculated based on the average expression of cytotoxic markers. P-value was calculated by a two-sided unpaired Kruskal-Wallis rank-sum test. (E) Sample distribution in each TILs subtype (colored according to group name).


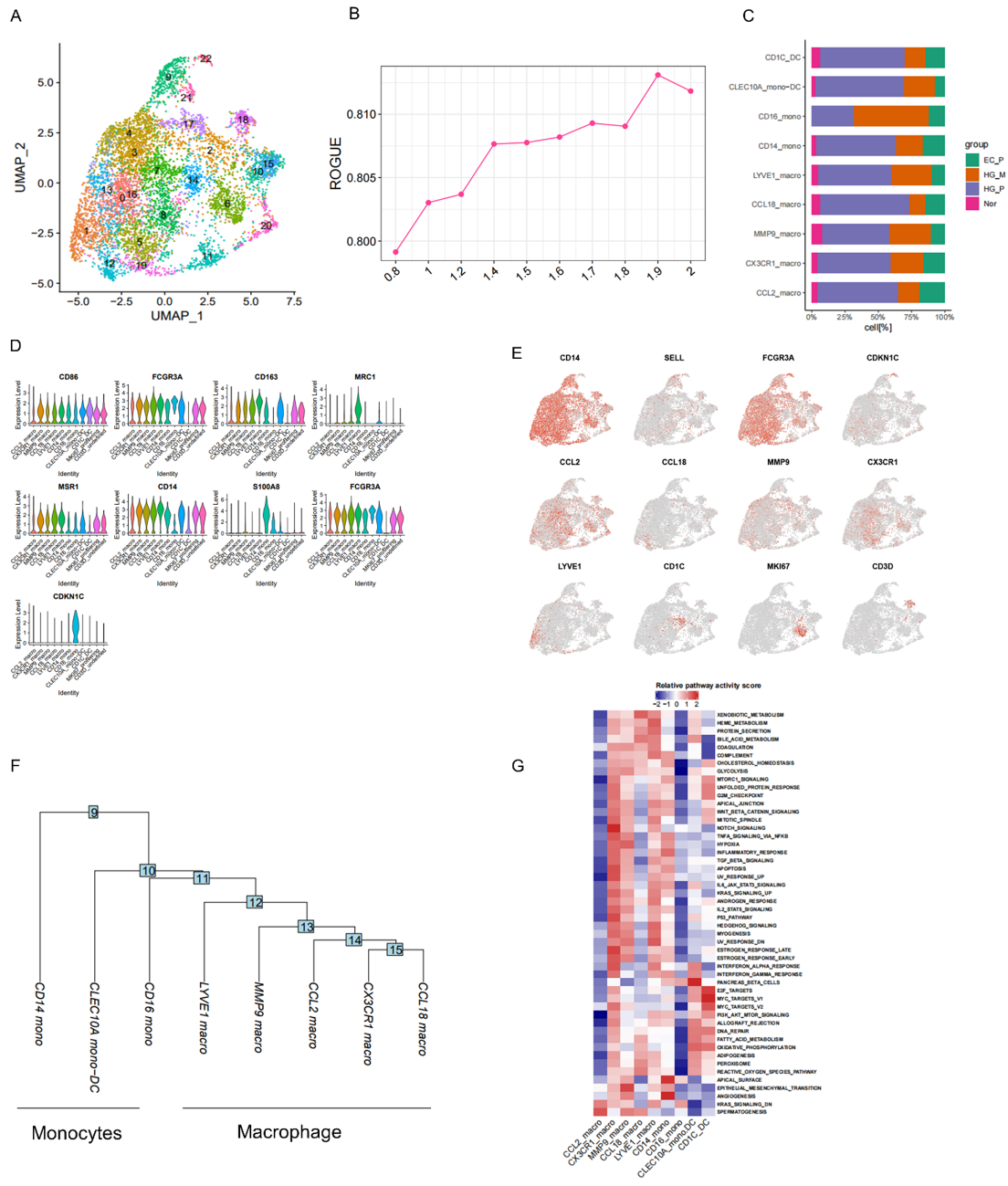


**Supplementary Figure 11.** Myeloid subtypes in ovarian lesions. (A) The UMAP projection of cell clusters in the myeloid population is predicted by the (B) ROGUE algorithm where the highest turning point represents the optimal value to assess the cell clusters. (D) The violin plot and (E) UMAP projection show canonical cell markers identified myeloid cell subtypes. (F) Phylogenetic analysis of myeloid subtypes using the BuildClusterTree function of the Seurat package. (G) GSVA analysis of differential pathways is scored per cell among each myeloid subtype.
